# A two-stage classification method for borehole-wall images with support vector machine

**DOI:** 10.1371/journal.pone.0199749

**Published:** 2018-06-28

**Authors:** Zhaopeng Deng, Maoyong Cao, Laxmisha Rai, Wei Gao

**Affiliations:** 1 Department of Electrical Engineering and Automation, Shandong University of Science and Technology, Qingdao, Shandong, China; 2 Department of Electronics, Communication and Physics, Shandong University of Science and Technology, Qingdao, Shandong, China; Nanjing University of Information Science and Technology, CHINA

## Abstract

Analyzing geological drilling hole images acquired by Axial View Panoramic Borehole Televiewer (APBT) is a key step to explore the geological structure in a geological exploration. Conventionally, the borehole images are examined by technicians, which is inefficient and subjective. In this paper, three dominant types of borehole-wall images on coal-rock mass structure, namely, border images, fracture images and intact rock mass images are mainly studied. The traditional image classification methods based on unified feature extraction algorithm and single classifier is not effect for the borehole images. Therefore, this paper proposes a novel two-stage classification approach to improve the classification performance of borehole images. In the first-stage classification, the border images are identified from three kinds of images based on texture features and gray-scale histograms features. For the remaining two types of images, in the second-stage classification, Gabor filter is first applied to segment the region of interest (ROI) (such as microfracture, absciss layer and horizontal cracks, etc.) and the central interference region. Then, using the same feature vector after eliminating the central interference region, fracture images are separated from intact rock mass images. We test our two-stage classification system with real borehole images. The results of experimental show that the two-stage classification method can effectively classify three major borehole-wall images with the correction rate of 95.55% in the first stage and 95% in the second stage.

## Introduction

The structural feature and mechanical property of fractures, absciss layers and other structural planes are significant to study the geological stability, engineering design and construction safety [1,2]. In geological exploration, the core boring method [3] is a traditional way to analyze the geological condition, which is characterized by heavy workload, low efficiency and difficulty in obtaining the cores of weak layers such as broken mudded intercalation and weathered interlayer. To overcome these shortages, Borehole Camera Technology (BCT) was introduced into the geological exploration in 1950s to directly observe the internal structure of geological bodies [4,5]. Thereafter, this technique has experienced about 3 phases, namely, Borehole Photo Camera(BPC), Borehole Televiewer (BTV) and Digital Borehole Optical Televiewer (DBOT). BPC uses photographic film to take static photos of the borehole-wall, which is lack of real-time monitoring capabilities. Currently, Axial View Panoramic Borehole Televiewer (APBT) [6] and Digital Panoramic Borehole Camera System (DPBCS) [7] are the most common techniques for the geological borehole observation. The DPBCS can obtain the section or entire of borehole-wall unrolled image, but the equipment is complex, expensive and only suitable for vertical holes [8, 9]. In contrast, the APBT can generate visualized panoramic images with simple structure, small volume and low cost. Moreover, it can be directly applied to horizontal holes and inclined holes, etc. [6].

Through borehole images acquired by APBT, we can observe the underground geological conditions. At the moment, however, these borehole images are usually examined by human eyes. It is time consuming and tedious to check possibly a large number of images even for experienced engineers. Moreover, it depends on the personal experience of engineers, which is weak in quantitative analysis and easy to cause errors. Therefore, it is necessary to establish some reliable and efficient approaches to analyze the borehole images. Recognition and classification of geological image have not been an object of active research in recent years although there have been some studies in this field. Khojasteh et al. [10] applies color and texture analysis for classification of keybeds in Gachsaran, and the upper Asmari formations and classification is done by using the SVM. Tools for classification were in that research co-occurrence matrix and fuzzy c-mean clustering (FCM). Jungmann et al. [11] successfully used the method of texture-based supervised classification to the classification of electrical borehole wall images. They extracted different texture features such as Haralick features, Zernike moments and wavelet-based features to combine with different classification methods and got a good classification results for certain rock groups. Yin et al. [12] analyzed the images characteristics of rock structure acquired by Formation Micro Imager (FMI) and developed a rock structure classification system. But its feature extraction method was simpler and coarse, which led to an unsatisfied classification accuracy. Although some researches have been conducted successfully on different types of geological image by image processing technology, they have not particularly focused on identification and classification of borehole images acquired by APBT. Therefore, this paper presents an automatic classification method for the borehole-wall images to assist geologists to survey and study for geological structure. We hope this has important practical significance for the geological engineering investigation and design.

Classification of geological images is an extremely difficult task in the field of visual inspection and image analysis [13]. In classification, a number of visual descriptors are extracted to classify images based on their content. The most common visual descriptors are colors, textures, and shapes occurring in the images. Nonetheless, the color of borehole-wall images is flat and unvaried, and the shape (such as fractures, joints, and structural plane, etc.) is usually irregular even if the images represent the same type. Thus these two features cannot discriminate the different classes best. Borehole-wall images can be classified into different categories based on their texture similarity [14]. Gray level co-occurrence matrix (GLCM) is probably the most popular method for texture analysis [15]. However, due to the similarity of horizontal crack images and intact rock mass images, a single statistical method of extracting the texture feature may not be sufficient for the classification task. Considering feature extraction and image recognition, many scholars have combined several algorithms for obtaining better results. Monika et al. [16] proposed that the variance of the GLCM combined with the normalized difference vegetation index (NDVI) is able to separate slums and formal areas. Park et al. [17] identified the candidate regions of ground glass opacity (GGO) based on homogeneity values calculated by the GLCM and the intensity values. Cheng [18] compared three feature extraction methods of intensity histogram, GLCM, and bag-of-words (BoW) model in the classification of brain tumors. Ashraf et al. [19] presented a reinterpretation of the application of Gabor filters, as a preprocessing step, to a linear SVM in terms of a manipulation of the margin. He et al. [20] proposed a fusion scheme to gain a better understanding and a fusion method for a face-iris-fingerprint multimodal biometric system. They used particle swarm optimization to train a set of adaptive Gabor filters in order to achieve the proper Gabor basic functions for each modality. In [21], GLCM and fractal features are extracted from the segmented ultrasound images to compose a feature space and classified using support vector machines (SVM) and artificial neural networks (ANN).

In this paper, we study borehole images with limited samples by using image processing and pattern recognition technologies. With analyzing the characteristics of borehole images obtained by APBT, we propose an automatic two-stage classification system to classify three dominant types of borehole-wall images, namely, border images, fracture images and intact rock mass images by using SVM [22], which replaces traditional classification method to improve the classification accuracy. Border images with relatively large effective regions have rich distinguishing features and are easy to be identified by the primitive image features, while fracture images and intact rock mass images have fewer features and are more complex. If the classifier is trained like the traditional method using three kinds of mixed samples, it may cause classification conflict, which will lead to unsatisfactory classification results. Therefore, in the first-stage classification, all three classes of image samples are mainly divided into border images and non-border images, and then the non-border images are put into the second stage among the classification of fracture images and intact rock mass images. Finally the classification results are merged together.

The rest of this work is organized as follows. Section 2 describes briefly the classification system framework. In Sec. 3, feature extraction, effective region segmentation and the used classifier are presented. Experimental results and some analysis are shown in Sec. 4, in which the proposed method is tested using real borehole images. Finally, Sec. 5 concludes this work.

## Proposed system

In this paper, we mainly concern our study on the classification of the most common three classes of borehole-wall images: border images, fracture images and intact rock mass images. In general, border images are characterized by large portion of bright area and clear contrasts across boundaries corresponding to a high degree of variability in the gray histogram, significantly different from other categories of images. Intact rock mass images are featured by highly homogenous in terms of directionality, granularity, and color. Typically, the differences between fracture images and intact rock mass images are not as clear as the border images. The fracture image is actually the intact rock mass image that exists fracture, abscission layer, and joint, etc. Therefore, the traditional classification methods that the features of all samples are extracted with a unified feature extraction method and then input into a single classifier did not allow us to distinguish the borehole images satisfactorily.

**Fig 1:**
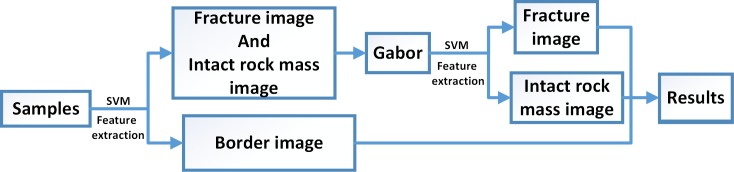
Two-stage classification method flowchart.

For the foregoing reasons, this paper presents a novel two-stage classification method to divide targets into three classes by two independent SVM classifier as shown in Fig 1, which can solve the above problem successfully. The specific process is as follows: In the first stage of our classification, based on texture features and gray features of original images, all pre-classified images should concurrently be made a binary decision: border image and non-border image. For the latter two classes, the visual differences of original images between the classes are not as clear as the first class. Hence, in the second-stage classification, we apply Gabor filter to effectively segment the region of interest (ROI) (such as absciss layer, horizontal cracks, etc.) and the central interference region, and then perform image segmentation to eliminate interference region. Finally, the processed images are divided into two types: fracture images or intact rock mass images. Our method constructs the two-stage classification model to enlarge differentiation advantage and gets the satisfactory classification results. The details of classification method is described in S1 File. And the relevant raw data are available in S2, S3 and S4 Files.

## Methodology

### Feature extraction

Feature extraction is a major part of image recognition, and it heavily affects the final classification accuracy. This study focuses on color analysis (gray analysis) and texture analysis to extract effective features from borehole-wall images. Furthermore, a SVM is utilized to classify the images into different types. Texture analysis methods have been utilized in a variety of application domains such as surface inspection, medical imaging and remote sensing, etc. [23]. Generally, there are lots of algorithms for texture feature extraction, including wavelet analysis, Laws texture extraction, Gabor filters, Local Binary Pattern (LBP), and GLCM. Admittedly, the GLCM is one of the main efficient methods of texture analysis. In the image classification, it is often beneficial to combine different visual descriptors to obtain the best possible classification result. In order to further improve the accuracy of classification, we introduce the statistical features of gray histogram for the classifier along with texture features.

### Texture feature extraction

Texture is one of the important features used in describing and assessing object surfaces [24]. Gray level co-occurrence matrix (GLCM) approach is a typical statistical analysis method, which is widely used in image textural analysis [25]. In this paper, we use GLCM as well to extract the texture features of borehole-wall images. It captures the degree of texture roughness and local variation of an image described by specific parameters. The local characteristics in the borehole-wall images, such as interface, cracks and absciss layer, are quantified by specific parameters defined by GLCM approach and used to recognize different categories of image.

Five statistical parameters of GLCM are taken into considerations. They are Angular Second Moment (ASM), Entropy (ENT), Inverse Difference Moment (IDM), Contrast (CON) and Correlation (COR), as shown below [26].

$$ASM=\sum_{i}\sum_{j}{P(i,j)}^{2}$$ (1)

$$ENT=-\sum_{i}\sum_{j}P(i,j)\log P(i,j)$$ (2)

$$IDM=\sum_{i}\sum_{j}\frac{1}{{1+(i-j)}^{2}}P(i,j)$$ (3)

$$CON=\sum_{i}\sum_{j}{\left(i-j\right)}^{2}P(i,j)$$ (4)

$$COR=\frac{\sum \sum \left(i-\bar{x})(j-\bar{y})P(i,j\right)}{\sigma_{x}\sigma_{y}}$$ (5)

### Gray-scale histogram feature extraction

In the process of capturing borehole images, as a result of the shooting environment, the borehole images are missing color information obviously, and thus the color feature should correspond to the gray feature. Feature extracting based on the gray histogram is a typical algorithm in the gray feature extraction of images. However, the histogram of image usually cannot be directly used as feature, but by statistical features of the image histogram, among which the most commonly used are gray mean and variance.

The gray mean reflects the average gray value of an image, which is defined as:

$$E=\sum_{i=1}^{m}\sum_{j=1}^{n}f(i,j)$$ (6)

and the gray variance indicates that the discrete distribution of image gray value, which is defined as:

$$V=\frac{1}{m\times n}\sum_{i=1}^{m}\sum_{j=1}^{n}{\left|f(i,j)-E\right|}^{2}$$ (7)

where *f* (*i*, *j*) is pixel gray value, and *m* and *n* defined the numbers of row and column in borehole-wall image.

**Fig 2:**
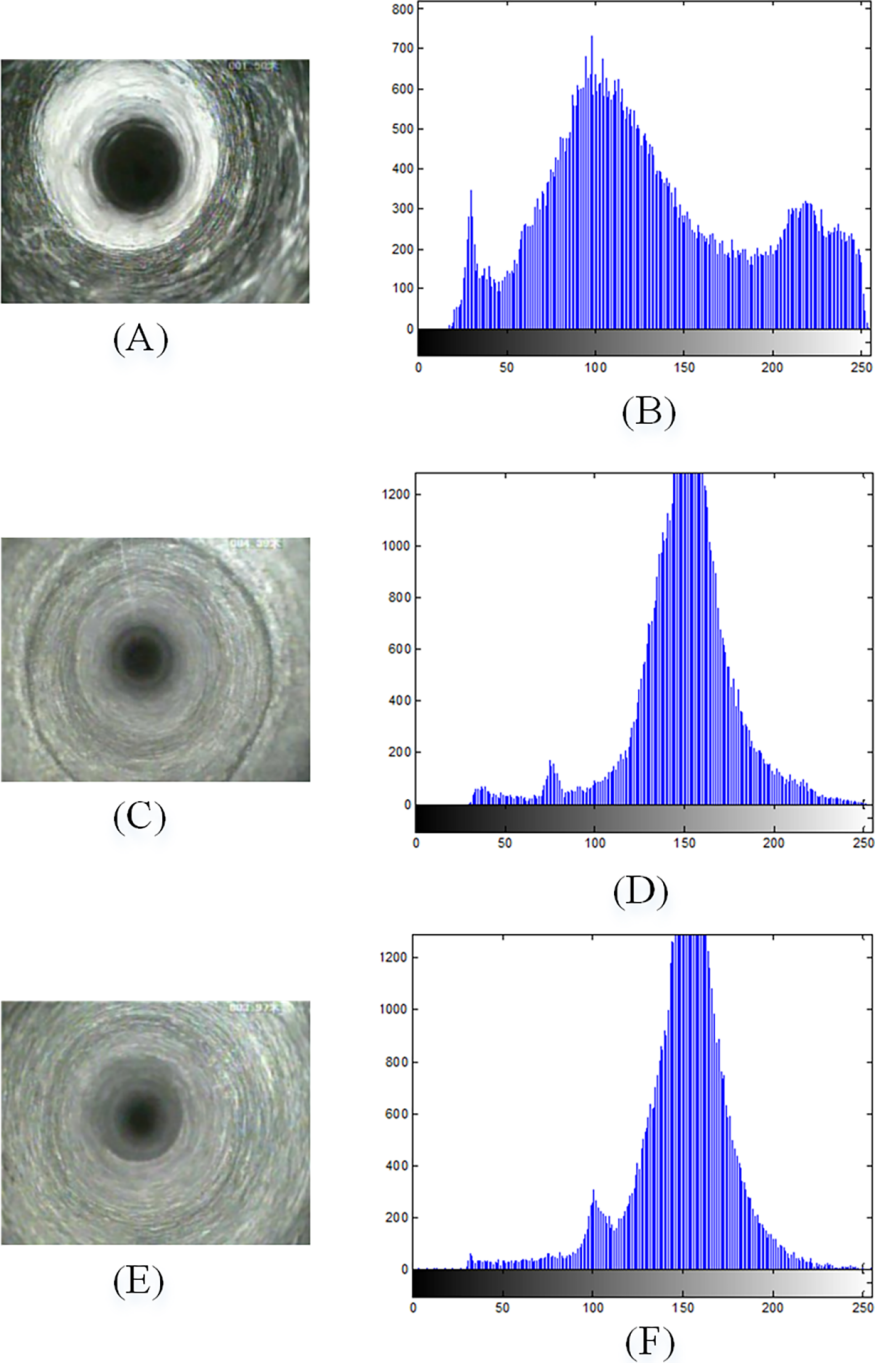
The gray histogram of original images.

In the first-stage classification of our system, we use gray-scale histogram features to separate border images from the other two classes of images. As shown in the Fig 2, the gray histogram of a border image (Fig 2B) has the obvious difference with the histograms of a fracture image and an intact rock mass image (Fig 2D and 2F), whereas the histograms of a fracture image and an intact rock mass image present a larger similarity. Therefore, statistical features of the gray histogram of original image can only be used as effective features for distinguishing border images from borehole images.

### Gabor transform

Gabor transform theory was proposed by Dennis Gabor in 1946 and later was extended to 2-D by Daugman [27]. In a 2D spatial domain, a Gabor wavelet is a complex exponential modulated by a Gaussian function, which can obtain high resolution in both time and frequency domains. The function can be defined as follows:

$$g(x,y,\theta ,f)=\frac{1}{2\pi \sigma_{x}\sigma_{y}}exp\left[-\frac{1}{2}(\frac{x_{0}^{2}}{\sigma_{x}^{2}}+\frac{y_{0}^{2}}{\sigma_{y}^{2}})\right]\exp\left[2\pi f\omega x_{0}\right]$$ (8)

where, *x*_0_ = *x* cos(*θ*) + *y* sin(*θ*), *y*_0_ = −*x* sin(*θ*) + *y* cos(*θ*), *x* and *y* denote the pixel positions, *σ*_*x*_ and *σ*_*y*_ are the variances of the Gaussian function along *x* orientation and *y* orientation respectively, *f* is the frequency of sine function, and *θ* represents the orientation of Gabor filter. By the experiments, when *f*, *σ*_*x*_, *σ*_*y*_, and *θ* are16, 2, 4, and *π*/3, respectively, the filtering effect of borehole images are the best.

Typically, an input image *I*(*x*, *y*) is convolved with a Gabor kernel *G*(*x*, *y*) to obtain a Gabor filtered image.

R(x,y)=G(x,y)*I(x,y) (9)

where, * is the convolution operator.

**Fig 3:**
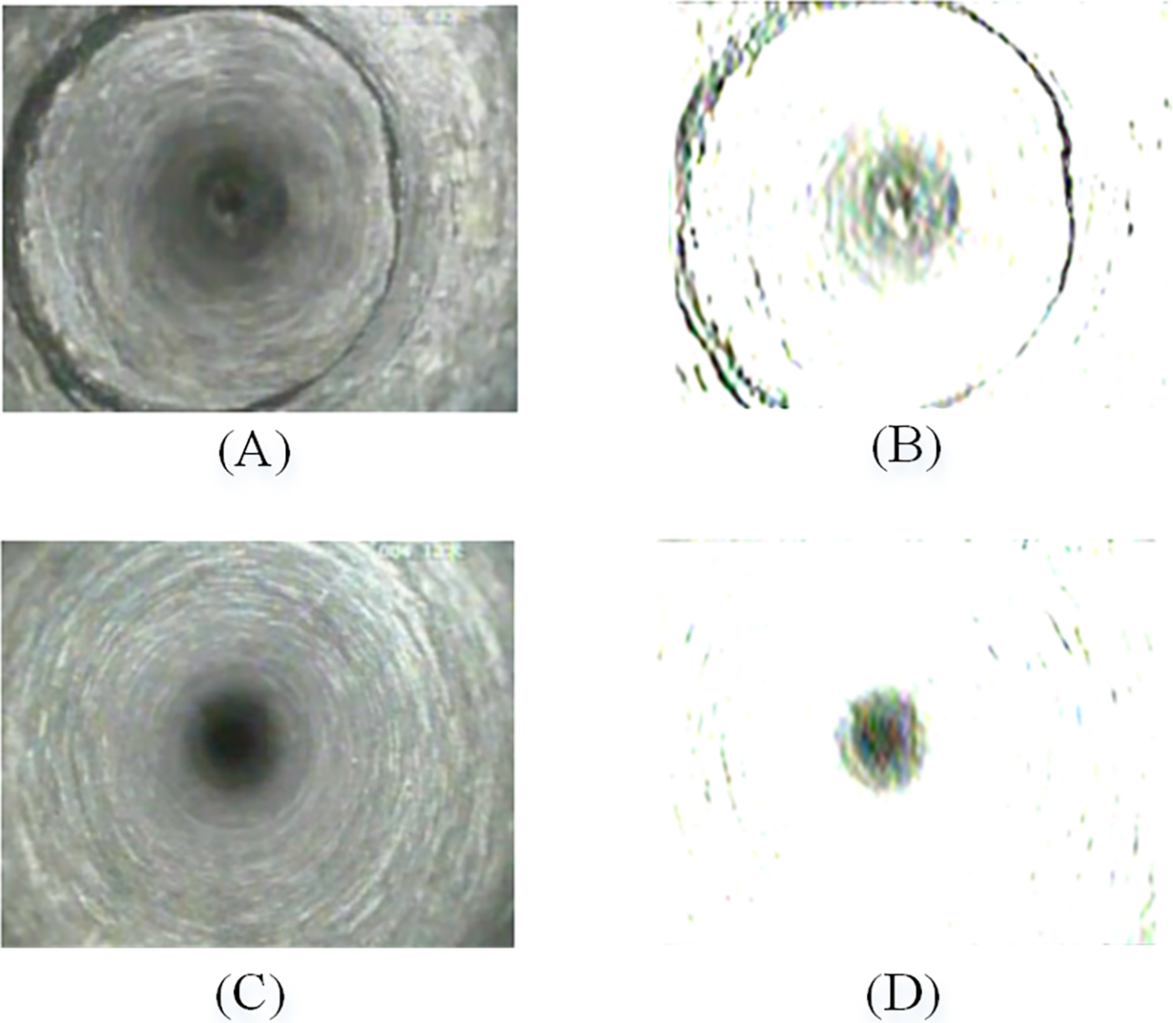
Gabor filtered effect. (**A**) fracture image; (**B**) Gabor filtered fracture image; (**C**) intact rock mass image; (**D**) Gabor filtered intact rock mass image.

Fig 3 shows an example of applying Gabor filter to a fracture image and an intact rock mass image. In Fig 3B and 3D, the filtered fracture image contains an absciss layer and a central interference region, and the filtered intact rock mass image only contains a central interference region. Although this method makes a large number of image information lost, it greatly expands the difference between fracture images and intact rock mass images, and therefore more discriminative features can be extracted to achieve a better classification.

### Image segmentation

**Fig 4:**
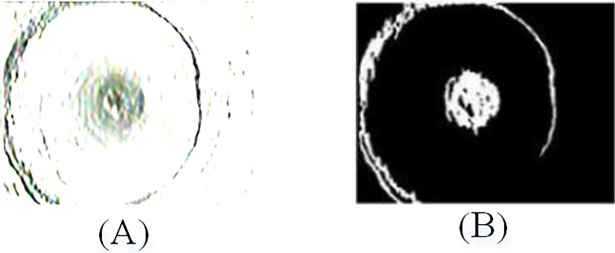
Gabor filtered image segmentation. (**A**) Gabor filtered image; (**B**) Threshold image.

Due to the imaging principle of axial view borehole TV, the center of the borehole image has a big visual blind spot (the central interference region), which is independent of the image content. As shown in Fig 3B, the center region and the fracture are segmented by the Gabor filter. The aim of image segmentation is to eliminate the central interference region. We found that the threshold value of 0.85 achieves best effective segmentation of the target region. Fig 4 shows an example of the image segmentation of a Gabor filtered image.

Usually, the central region has two common features: the area is almost the biggest in all regions, and the shape is closer to circle. Hence, the area and circularity can be combined to achieve the location of the central interference region. The region-labeling algorithm is used to assign the same mark to each connected region. And the area of each region, defined as the number of pixels in the region, are calculated. The circularity is generally defined as follows:

$$e=\frac{4\times \pi \times \alpha }{p^{2}}$$ (10)

where *α* is the area of region, *p* is the border length of region, and *e* represents the similarity between the region and circular.

**Fig 5:**
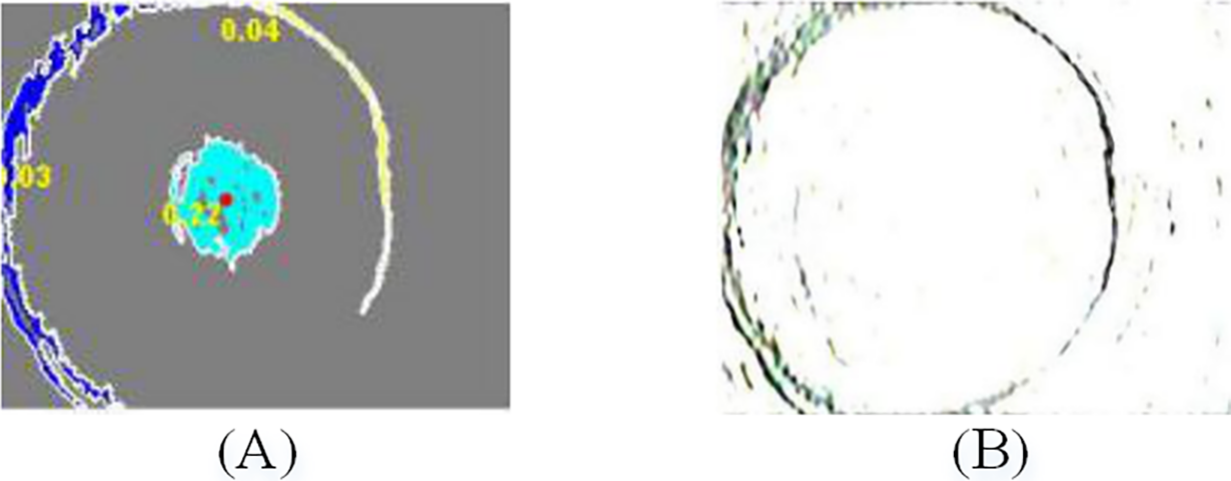
Eliminate center region. (**A**) Region labeling; (**B**) Eliminate center interference region.

In our borehole-wall images, the region of the largest area and circularity identify the central interference region. As shown in Fig 5A, the center area contains 2868 pixels and circularity is 0.22, both are the maximal values in all regions. Therefore, we can locate the centroid of central region and eliminate the interference region within an appropriate range, as is shown in Fig 5B.

### SVM classifier

**Fig 6:**
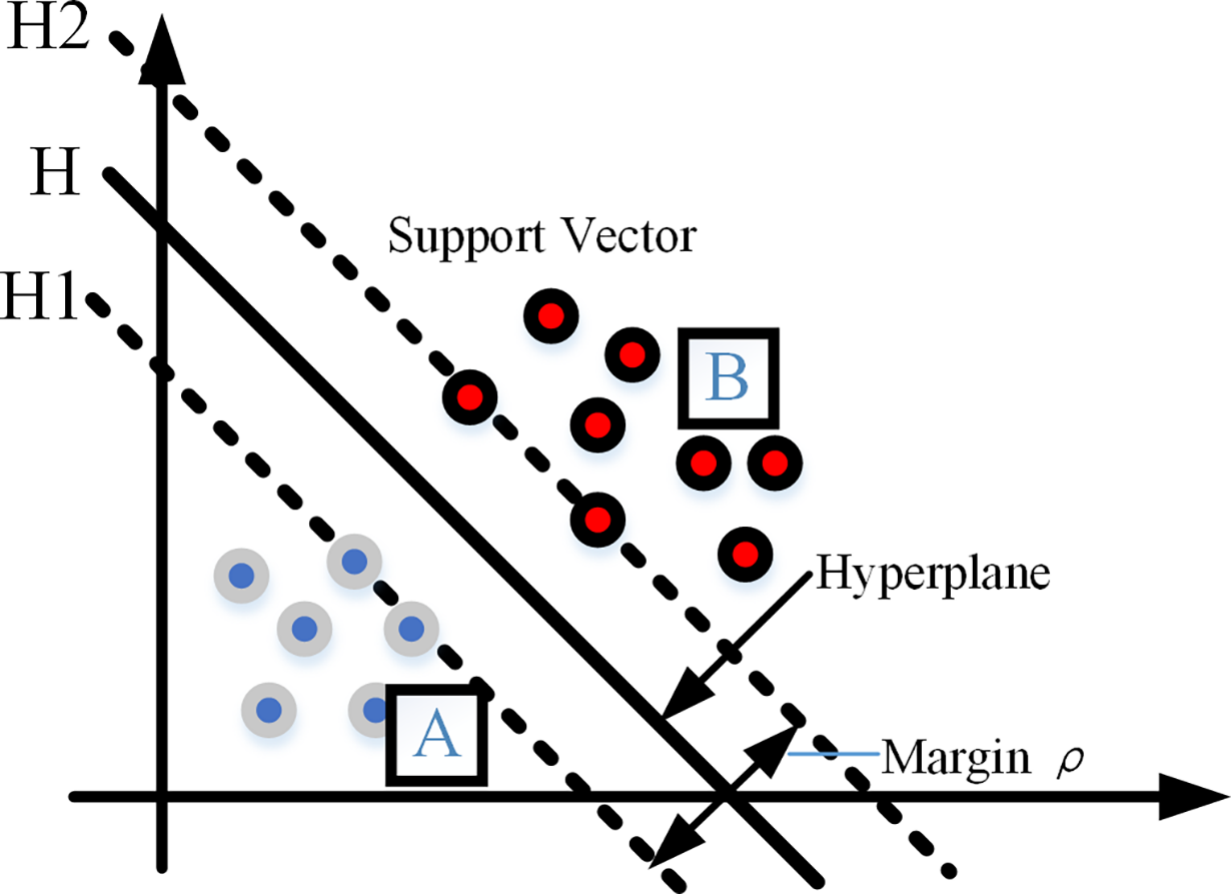
Optimal separation hyperplane.

Support Vector Machine (SVM) classifier, based on statistical learning theory and structural risk minimum principle, is a new machine learning classification algorithm [28,29]. The main aim of SVM is to obtain the decision boundary or hyperplane which optimally separates two kinds of samples as illustrated in Fig 6. Where *H* is the hyperplane, *H*_*1*_ and *H*_*2*_ are planes parallel to the hyperplane, the distance ($\rho =\frac{2}{\left\|\omega \right\|}$) between *H*_*1*_ and *H*_*2*_ is the separating margin, and *ω* is a vector defining the boundary. For a given training set, seeking an optimal hyperplane is to maximize the separating margin between two classes [30].

When samples are non-linear, quadratic programming method is used to obtain optimal hyperplane, as shown in formula (11):

$$\left\{ \begin{array}{c} \emptyset (\omega )=\frac{1}{2}{\left\|\omega \right\|}^{2}+C\sum_{i=1}^{N}\varepsilon_{i}\\ y_{i}[(\omega^{T}Z_{i})+b]-1\geq 0,i=1,2,\cdots ,N \end{array}\right.$$ (11)

where ∅(*ω*) is object function, *ε*_*i*_ is slack variable, and *C* is penalty factor. The optimal classification function is as follows.

$$f(x)=sign(\sum_{i-1}^{n}\alpha_{i}y_{i}\langle x,x_{i}\rangle +b)$$ (12)

When we solve the optimization problem of SVM, kernel function *K*(*x*, *x*_*i*_) based on the Mercer’s theorem can replace inner product ⟨*x*,*x*_*i*_⟩, which implicitly makes the input vector map into a high-dimensional feature space, thus the nonlinear problem can be solved as a linear problem.

There are several types of kernel functions, namely, liner kernel function, polynomial kernel function, radial basic kernel function (RBF) and Sigmoid kernel function. The decision function can be expressed as follows:

$$f(x)=sign(\sum_{i-1}^{n}y_{i}\alpha_{i}K\langle x,x_{i}\rangle +b)$$ (13)

where *α*_*i*_ and *y*_*i*_ are Lagrange multipliers, and *x*_*i*_ = [*x*_1_, *x*_2_, *x*_3_,…, *x*_*n*_] is the input data.

### Classification experiments with borehole-wall images

In this section, the performance of the proposed two-stage classification method is examined using the borehole images acquired by Axial View Panoramic Borehole Televiewer (APBT). In all experiments, SVM is selected to be the classifier for supervised classification, which is suitable for small sample classification.

### The database of borehole-wall images

**Fig 7:**
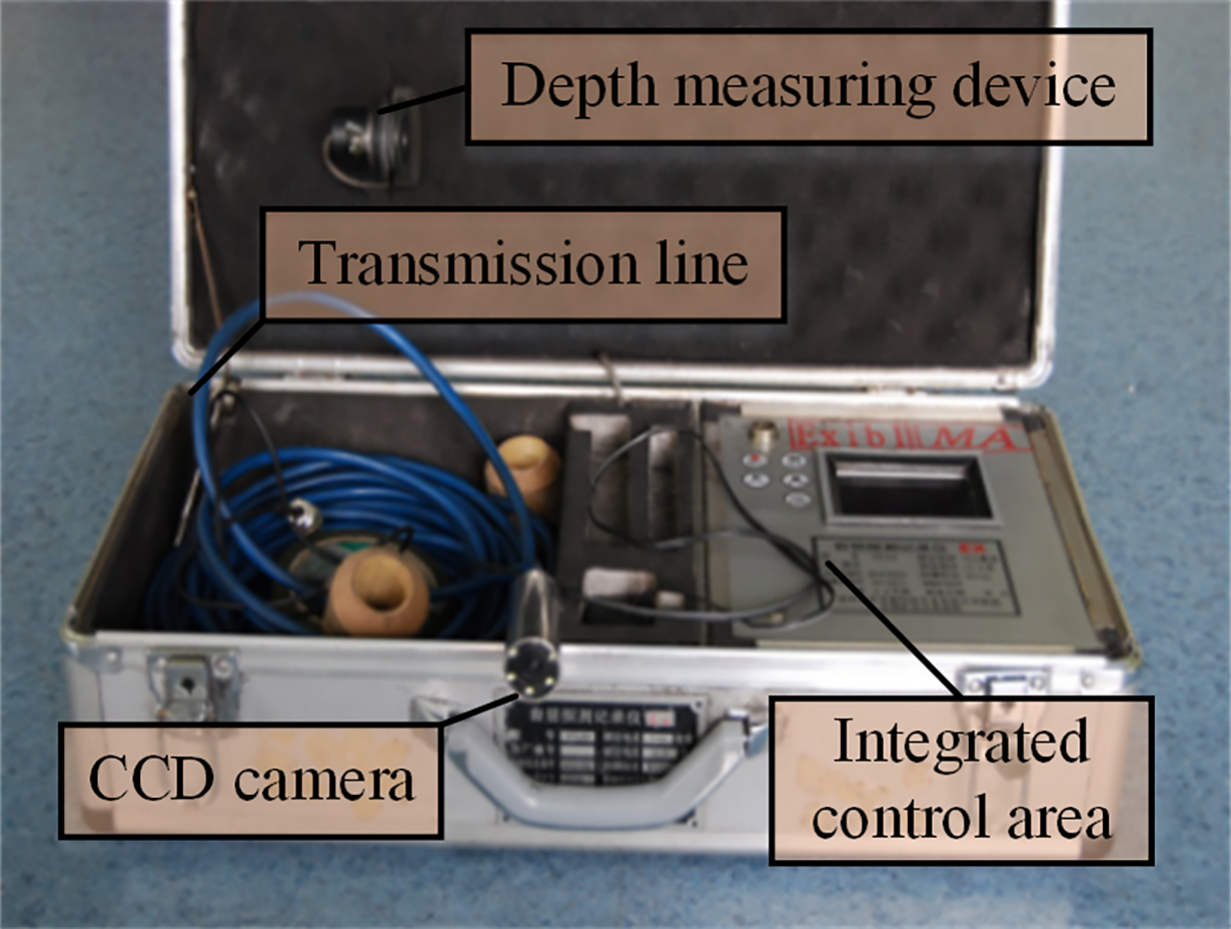
Components of an APBT system.

The APBT can directly observe the structural feature and mechanical property of rock mass through pre-drilled borehole, with characteristics of the borehole-wall being surveyed in air or clear fluid filled boreholes. We adopt the YTJ20 type of APBT system, which mainly consists of the CCD camera, transmission line, guide bars, depth measuring device, integrated control box as shown in Fig 7, and its specifications are given in Table 1. The resolution of the image acquired by this apparatus is up to 0.1 mm, which can observe the distribution of small fractures in borehole.

**Table 1:** Main parameters of YTJ20 type APBT.

Probe diameter	Probe length	Size of Host Machine (Length×Width×Height)	Continuous operating time	Storage capacity of video
25 (mm)	100 (mm)	240×190×83 (mm^3^)	8 (h)	2 (G)

**Fig 8:**
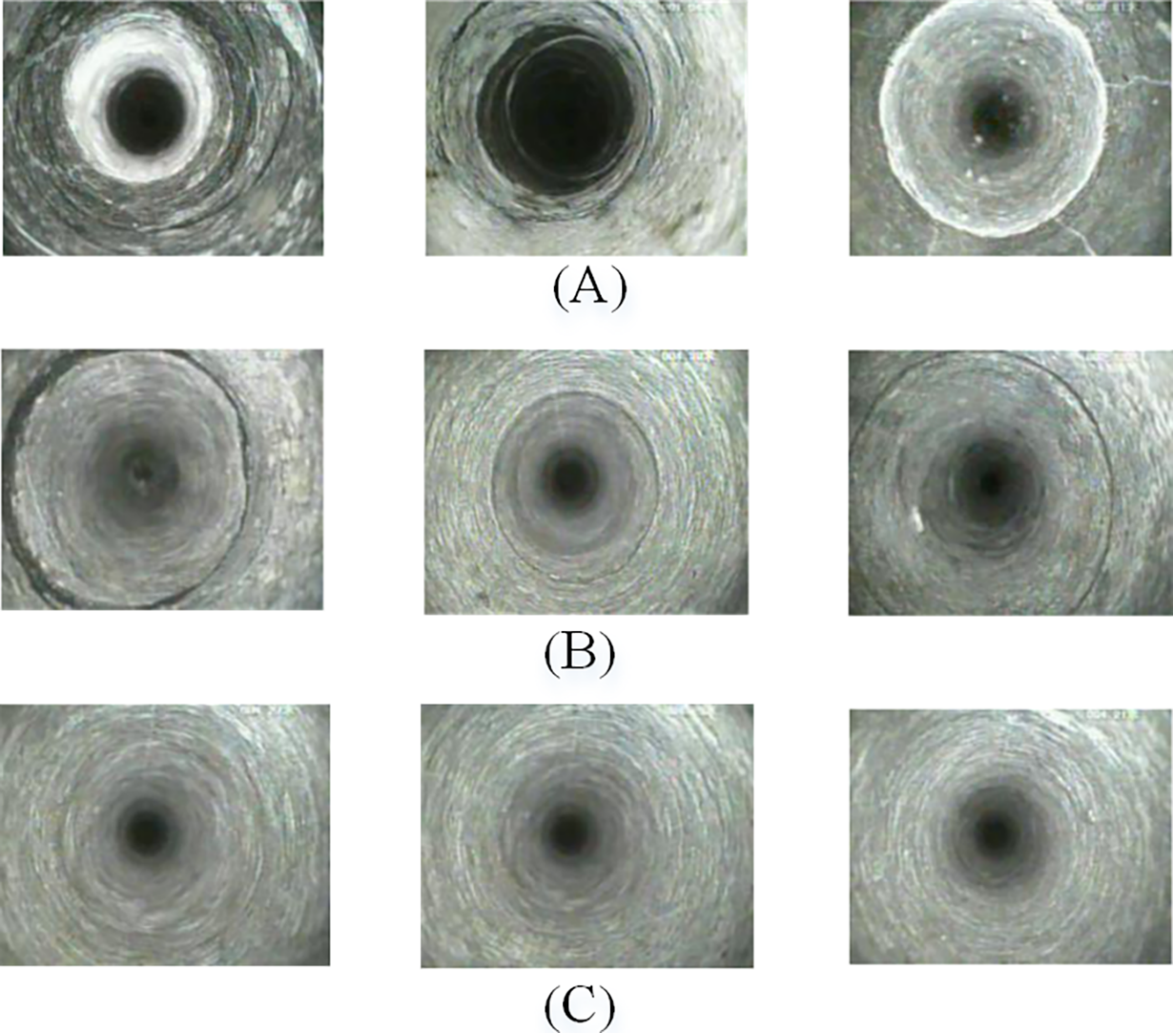
Samples of borehole-wall images. (**A**) border images; (**B**) fracture images; (**C**) intact rock mass images.

The borehole-wall image samples for this research are obtained from the coal mine exploration [31]. These images are manually divided into three classes by an experienced geological expert as: (1) border images, including coal-rock boundary and different rock boundary, etc. (2) fracture images, including absciss layer, microfracture, joint and cracks, etc. and (3) intact rock mass images. The division is based on their color and texture properties. There are total 150 original image samples and each of the three classes contains 50 images where 30 images are randomly selected as training images and 20 images as testing samples. The size of the each image is 300 × 238 pixels. Fig 8 presents three example images of each three class. The objective of these experiments is to make a classification between these image classes.

### Feature extraction and normalization

In the classification experiments, different visual descriptors are extracted from the database of borehole-wall images. We use two descriptors: Gray level co-occurrence matrix (GLCM) with five features and two statistical features of the image histogram.

For the textural feature extraction of borehole-wall image, we performed a texture analysis to create the feature vector of each image, which is composed by five GLCM features in four directions (0°, 45°, 90°, 135°): ASM, ENT, IDM, CON and COR. These features are simply and fast computed based on the co-occurrence matrix and have been demonstrated to be very discriminative in the image classification. In order to improve the robustness of parameter to the direction, the average value of four directions is taken by the formula (14). The extracted texture feature vector is (*ASM*, *ENT*, *IDM*, *CON*, *COR*).

$$f=(f_{0}+f_{45}+f_{90}+f_{135})/4$$ (14)

Afterwards, the mean and variance of image are calculated by using formula (6) and (7) respectively to compose the gray feature vector. For each sampling image, texture features combined with gray features form a seven-dimensional feature vector ***x*** *=* (*ASM*, *ENT*, *IDM*, *CON*, *COR*, *E*, *D*), which is labelled with the corresponding borehole-wall image class.

To ensure that the data is in same quantity rank, normalized method is adopted to pretreat the imported data during training and testing the SVM classifier processes. Formula (15) is the data normalization model.

$$\bar{x}=(x-x_{min})/(x_{max}-x_{min})$$ (15)

where *x* is the raw data, *x*_*max*_ and *x*_*min*_ are the maximal and minimal values of data, respectively.

## Experimental results and analysis

To ensure the validity of the proposed system, this section makes three experiments, which demonstrate the effectiveness of the two-stage classification model and Gabor filter. And moreover, we compare the performances of different classifiers and filtering algorithms in classification of borehole images.

### Experiment (1)

The innovation of traditional classification methods mainly lies in the image feature extraction or the classifier optimization, using a unified feature extraction method for samples of all classes and a single classifier. To confirm the validity of two-stage structure, traditional image classification methods by using one classifier are done and then different kernel functions for evaluating the performance of SVM are tested. In the first experiment, we directly extract the features of all three types of images for classification without the method of two-stage, and choose three different feature extraction methods:

Algorithm (1): In this method, five texture features of original images extracted by GLCM combined with two gray features constitute the feature vector, which is selected as an input for the SVM classifier.
Algorithm (2): In this case, the borehole-wall images are decomposed by multi-scale wavelet, then the energy and moments as the features to be recognized by SVM are extracted.
Algorithm (3): The second-stage classification method in this paper is applied to classify all three types of images. All kinds of borehole images are filtered by Gabor and eliminated the central interference region, then the same seven-dimensional feature vector is extracted and inputted to the SVM classifier.

**Fig 9:**
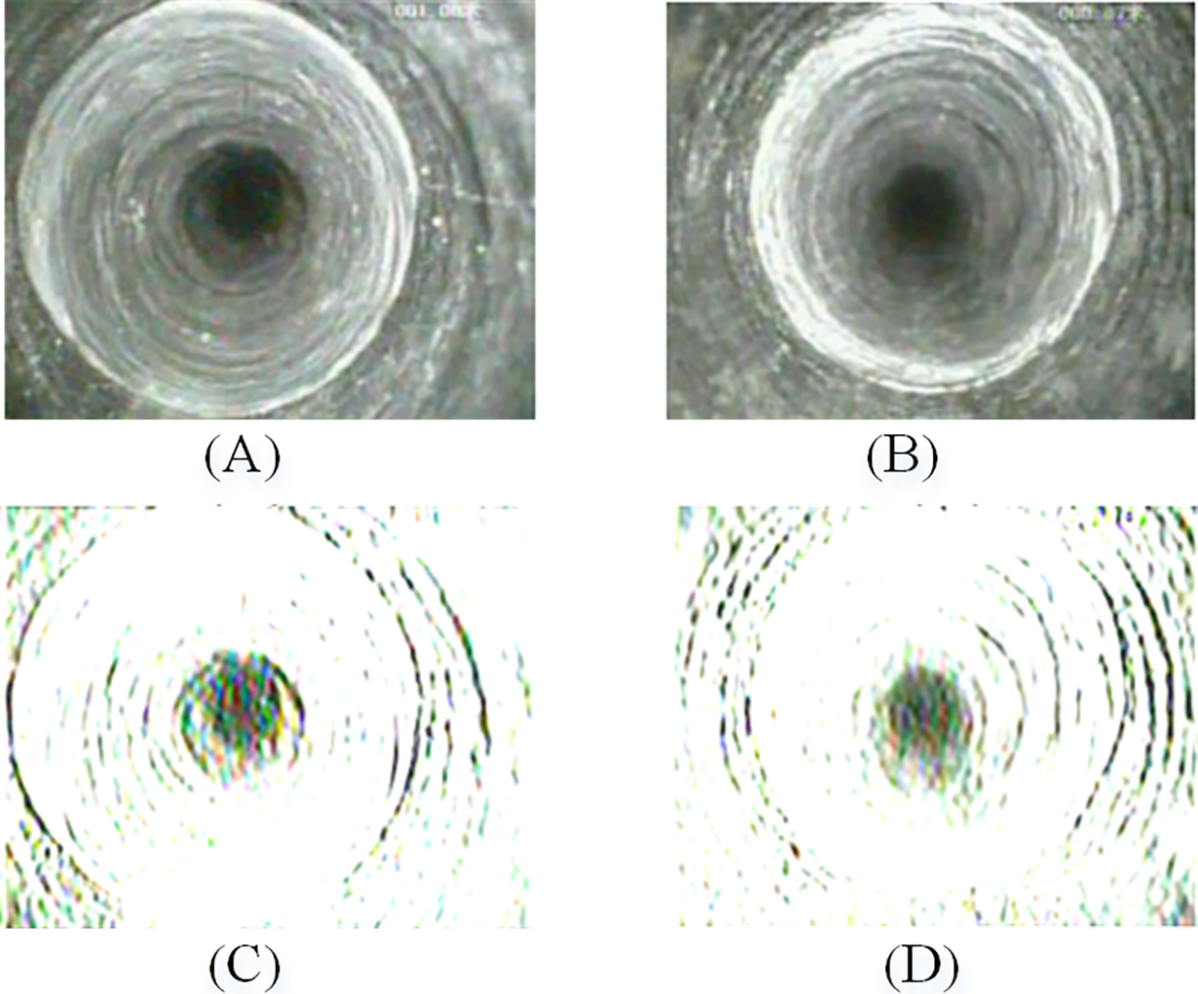
Gabor filtered images. (A) and (B) are original border images; (C) and (D) are Gabor filtered border images.

However, as shown in Fig 9, much information which is essential for distinguishing border images from the others will be lost. Moreover, after the Gabor filtering, some of the border images are similar to the fracture images, thus increasing the difficulty in classification. Fig 9C and 9D are Gabor filtered border images which similar to the Gabor filtered fracture images.

The accuracy of algorithm (1), (2) and (3) are shown in Table 2. As can be seen from Table 2, a single statistical method of GLCM has the highest accuracy. Since distinguishing features of border images are lost, algorithm (3) cannot work well for classification of all borehole-wall images.

**Table 2:** The accuracy of traditional image classification methods.

Method	Total number of images	Number of images in training	Number of images in testing	Accuracy of training samples (%)	Accuracy of testing samples (%)
**Algorithm_(1)**	150	90	60	83.33	80.00
**Algorithm_(2)**	150	90	60	76.66	71.66
**Algorithm_(3)**	150	90	60	77.78	75.00

**Fig 10:**
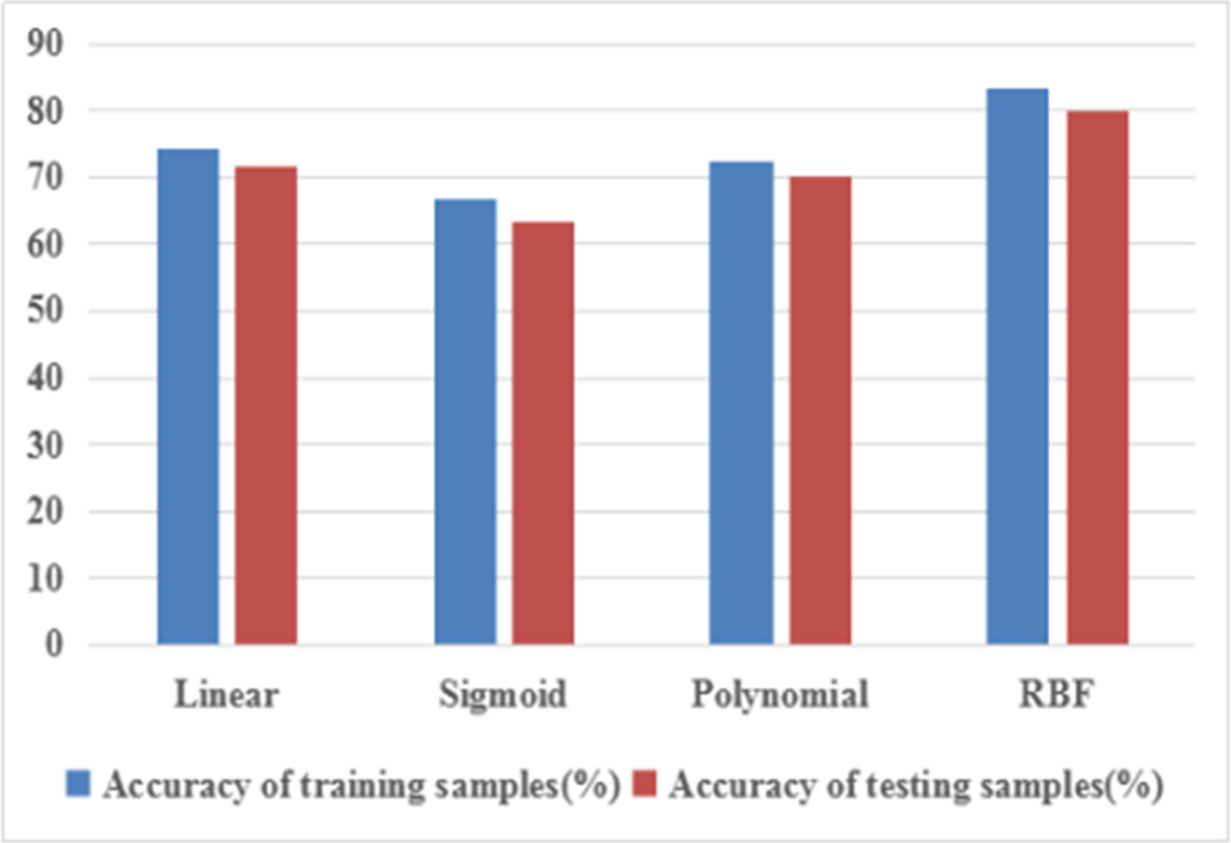
Classification accuracy of different kernel functions.

The selection of the kernel function will affect the precision of the SVM [32]. Until now, there is no effective method to select an optimal kernel function for a particular question. Therefore, different kernel functions for evaluating the performance of SVM are tested. Algorithm (1) is used for the choice of optimal kernel function, and the accuracy is shown in Fig 10. It can be seen that RBF is the most successful classifier in distinguishing borehole images with 83.33% accuracy in comparison to the Linear, Sigmoid and Polynomial with 71.66%, 63.33% and 70% (testing samples), respectively. Therefore, in this paper, RBF is used as the kernel function for SVM.

### Experiment (2)

In this section we have illustrated the validity of these texture and gray features in distinguishing between the samples of fracture images and intact rock mass images after using Gabor filter and image segmentation. In our second-stage classification, the method combining Gabor filter and image segmentation technology is used to improve the distinguish capability of features in latter two classes.

**Fig 11:**
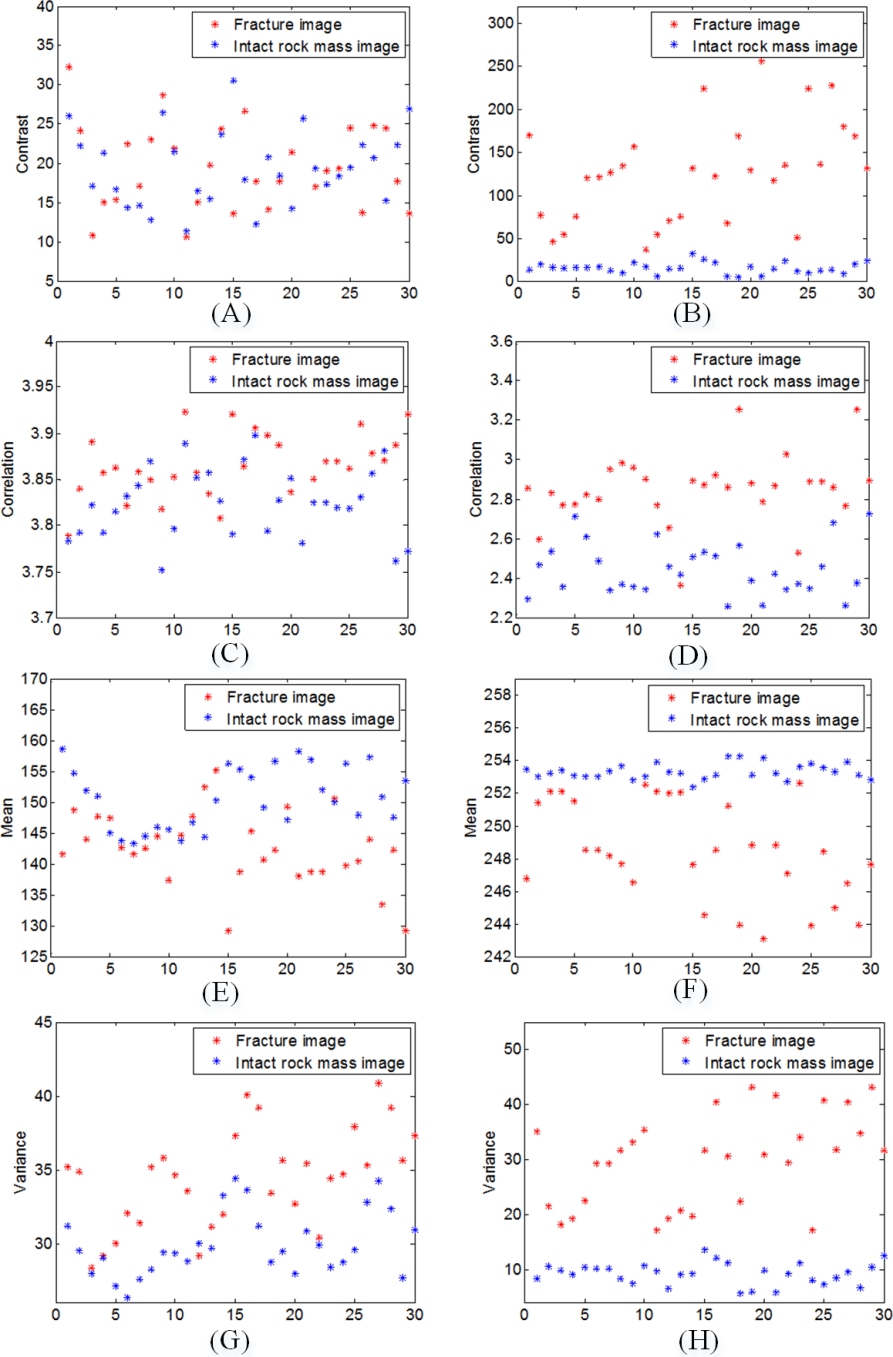
Discriminative ability of features. (**A**) contrast of original images; (**B**) contrast of processed images; (**C**) correlation of original images; (**D**) correlation of processed images; (**E**) mean of original images; (**F**) mean of processed images; (**G**) variance of original images; (**H**) variance of processed images.

Fig 11, in which the horizontal axis is the samples number and the vertical axis describe feature value, shows different characteristics of each class conferring to different features of GLCM and gray value. The discriminative ability of contrast is shown in Fig 11A and 11B, presenting its value for 30 images in the fracture image class and intact rock mass image class. It can be seen from Fig 11A that these values are interlocked together, and thus cannot distinguish the two types of images. Remarkably, after the Gabor transform and image segmentation, Fig 11B illustrates that the contrast values between two classes have big difference and the gap is wide in comparison to the original feature values (in Fig 11A), which make the contrast become an effective distinguishing feature for image classification. This is quite obvious, using Gabor filter to the remaining two classes is due to the fact that the processed image can highlight the ROI (such as absciss layer, microfracture, and joint etc.), and the central interference region can be removed. The result can be observed in Fig 11B, where samples of the fracture images are located at high values and the intact rock mass images show small values. The distribution of these two separated clusters presented gives evidence that a decision boundary can be established with good discrimination and, consequently, low probability of classification error.

Subsequently, Fig 11C to 11H show the contrast effects of correlation, mean, and variance, respectively. Consistent with this observation, a SVM classifier shows the best performance for this task with 95% of accuracy for the latter two kinds of borehole images. This performance supports that the proposed second-stage classification method is effective, and thus can differentiate fracture image and intact rock mass image accurately.

### Experiment (3)

In the borehole image classification, the classification accuracy of different classifier is different. It is related with statistical distribution characteristics of data, prior knowledge, the size of samples and structure of classifier itself and so on. In the third experiment, we compare the support vector machine (SVM) [33] and artificial neural network (ANN) classifiers for the classification of borehole images, and analyze the filter performance of wavelet filtering instead of Gabor in the second-stage classification. The third experiments use the two-stage classification approach, thus the first-stage classification have the same accuracy which is the recognition rates of border images. The experiments are as follows:

Algorithm (4): In this method, the low-frequency coefficients image of wavelet decomposition are used to replace the Gabor filtered image in second-stage classification.

Proposed method (ANN): In this case, two classifiers in our proposed method are replaced by ANN classifier.

For classifier, the LibSVM [34] with RBF kernel and the Fast Artificial Neural Network Library (FANN) [35] are employed. The parameter setting of FANN is *n*:*y*:*c*, where *n* (number of features), y = |Z_1_|-1 and *c* (number of classes) are the number of neurons in the input, hidden and output layers, respectively.

**Fig 12:**
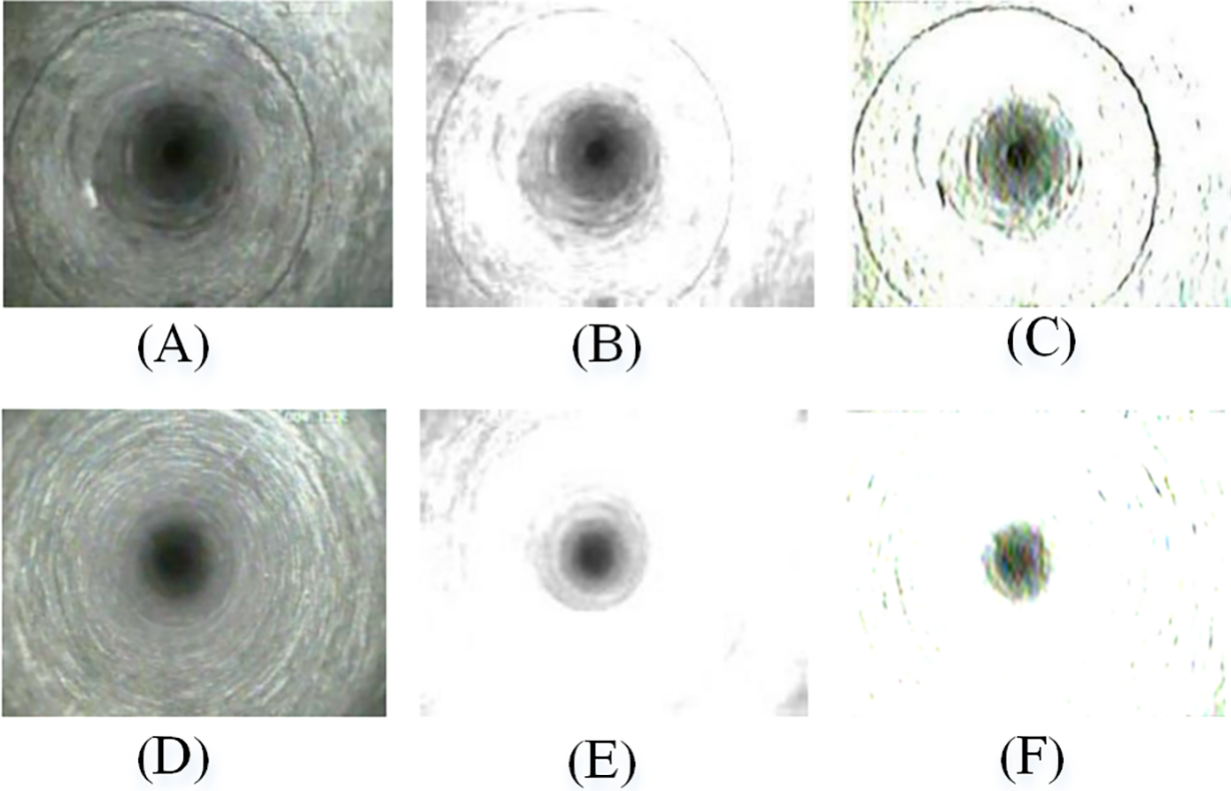
Wavelet image and Gabor filtered image. (**A**) and (**D**) are original images; (**B**) and (**E**) are low-frequency images of wavelet decomposition; (**C**) and (**F**) are Gabor filtered images.

The first-stage classification accuracy is the recognition rate of border images, which is higher than that of the traditional image classification methods, as shown in Table 3. Both the wavelet transform and Gabor filter can reflect the local detail information of the image in each scale [36,37]. Due to the application of Gabor filtering, we particularly improved the classification effect for the fracture image and intact rock mass image class, the most problematic classes of the borehole image classification. The classification accuracy of Gabor reached 92.5% while wavelet transform it correctly classify the test set at a rate of 87.5%. From the result above, it is found that the classification effect of Gabor filter is better than wavelet transform mainly. Because after wavelet decomposition, the low-frequency coefficients of the image is not clear in the ROI, and the outer edge exists a lot of interference. By contrast, Gabor filter effectively eliminates the noise region and segments the ROI better [38]. The filtering results of the two algorithms are shown in Fig 12.

**Table 3:** Classification accuracy of algorithm 3 and this paper.

Method	First-stage accuracy (%)		Second-stage accuracy (%)	
	Training samples	Testing sample	Training samples	Testing sample
**Algorithm_(4) **	95.55	93.33	85.00	75.00
**Proposed method_(ANN)**	95.55	93.33	91.66	87.50
**Proposed method_(SVM)**	95.55	93.33	95.00	92.50

**Fig 13:**
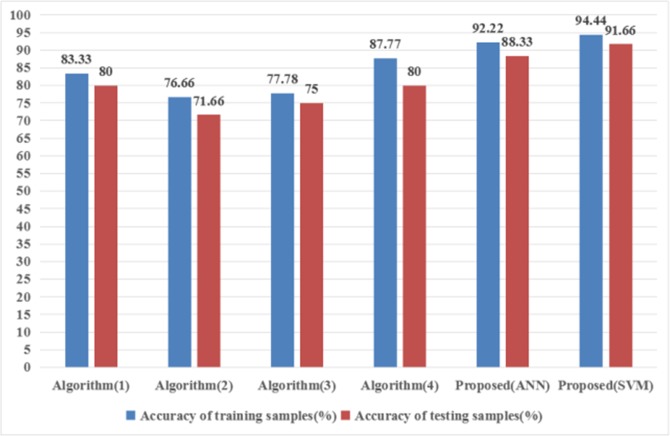
Classification accuracy of different methods.

The obtained results in Fig 13 show that the two-stage classification method based on SVM is able to outperform the conventional image classification strategies in the classification of borehole images. And as the limited of image samples, SVM classifier has a greater improvement than ANN classifiers. The accuracy of the proposed method is able to achieve 94.44% in training samples, thus it shows that the algorithm proposed in this paper is effective.

## Conclusion

Analyzing images of geological drilling holes is an important and crucial task to explore the geological structure. Several studies have been developed for geological image analysis, but few of them take care about identification and classification of borehole images obtained by Axial View Panoramic Borehole Televiewer (APBT). Therefore, this paper presents a novel two-stage classification approach for the automatic classification of borehole images. It can improve the classification accuracy of borehole images significantly. At the first stage of classification, the border images are recognized by the first level SVM from three types of borehole images based on texture and gray features of original image. Afterwards, in the second-stage classification, the ROI of the fracture images and intact rock mass images are extracted by Gabor filter and image segmentation technology, and then the processed images are well classified by the second SVM.

Experiments with real borehole images captured from the coal and rock exploration show that the proposed two-stage classification method is more effective than the traditional method of classification since it highlights the differences between the fracture images and intact rock mass images, and consequently extract more discriminative features. The proposed method gives promising results in classification of the borehole-wall images by using SVM classifier with RBF kernel and the results of this research would be highly helpful in analyzing images of geological drilling holes. On the test set, the classification accuracy in the first-stage and second-stage has reached 93.33% and 92.5% respectively.

Our classification system is with very limited samples due to the strict conditions and practical limitations. A higher accuracy is expected if more samples are given. Future work involve expanding the number of image samples and also selecting more and better features to perform the classification task.

## Supporting information

S1 File Detailed description of methods used for data processing and analysis. (PDF)

S2 File Original data for the border image. (ZIP)

S3 File Original data for the fracture image. (ZIP)

S4 File Original data for the intact rock mass image. (ZIP)
