# Supplementary material for: A two-stage classification method for borehole-wall images with support vector machine
Source: PLoS One. 2018 Jun 28;13(6):e0199749. doi: 10.1371/journal.pone.0199749 (PMC6023159; doi:10.1371/journal.pone.0199749)
Supplement: S1 File — (PDF) [file pone.0199749.s001.pdf]

There are total 150 original image samples and each of the three classes contains 50 images where 30 images are randomly selected as training images and 20 images as testing samples.

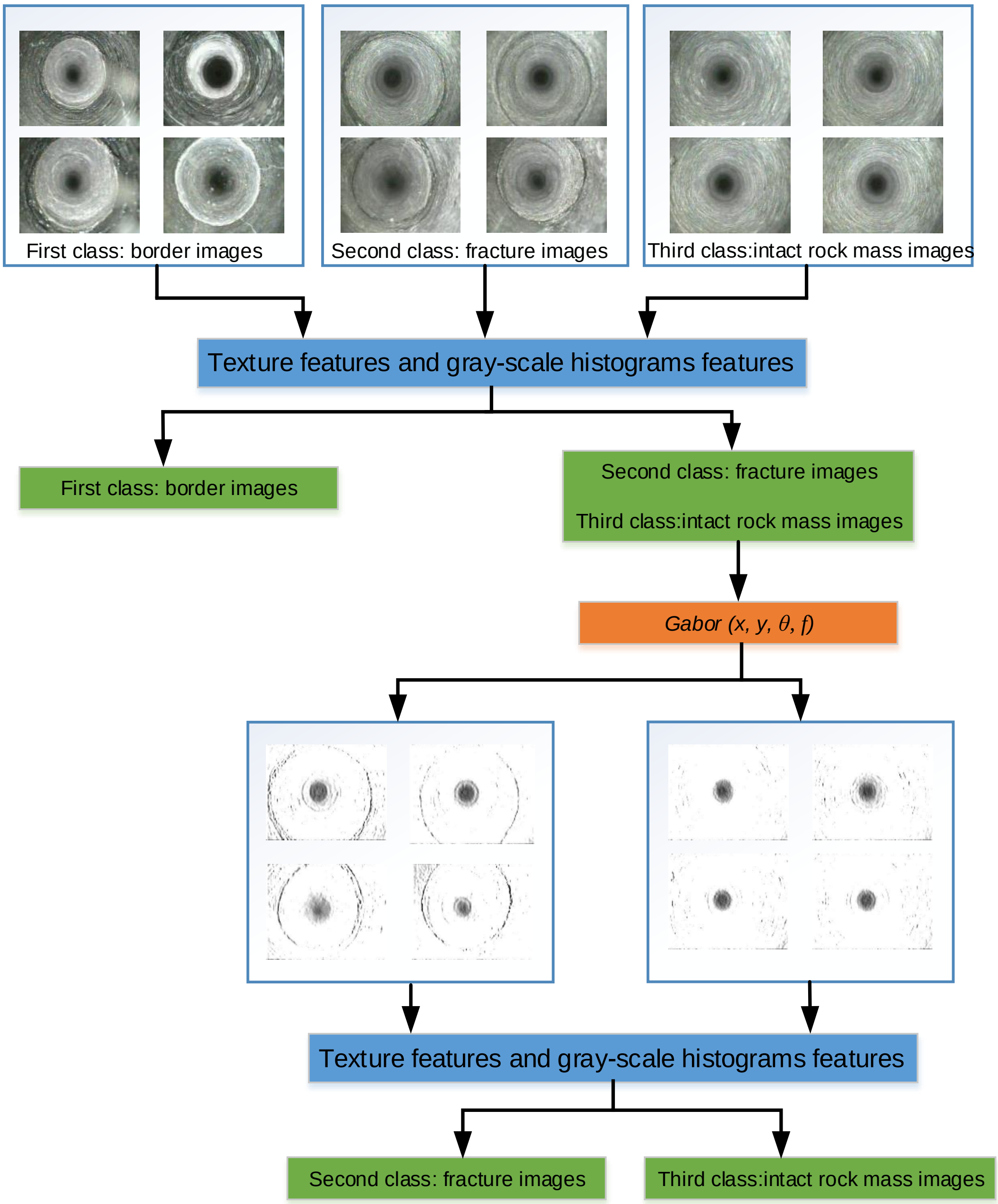

In the second-stage classification, Gabor filter is applied to segment the region of interest and the central interference region. Then, using the same feature vector after eliminating the central interference region, fracture images are separated from intact rock mass images. However, some fracture images are similar to the intact rock mass images. As shown in the following figures, these two fracture images are identified to the intact rock mass images after Gabor filter.

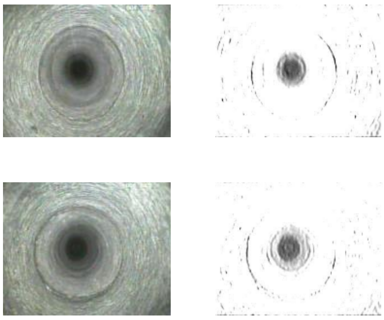

In contrast experiments, the borehole-wall images are decomposed by multi-scale wavelet, some images are shown as follows.

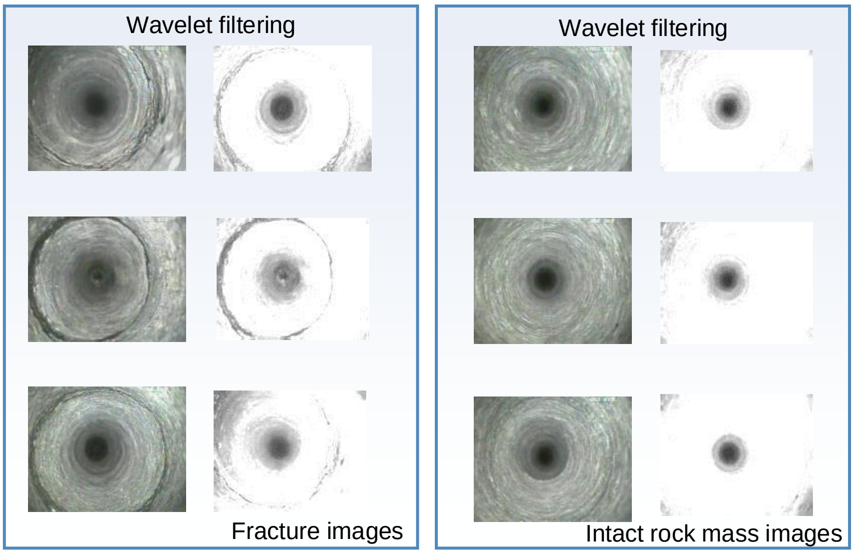

If image data is required, please contact through e-mail: my-cao@263.net
