# Supplementary figures and images for: A two-stage classification method for borehole-wall images with support vector machine (part 1 of 2)
Source: PLoS One. 2018 Jun 28;13(6):e0199749. doi: 10.1371/journal.pone.0199749 (PMC6023159; doi:10.1371/journal.pone.0199749)

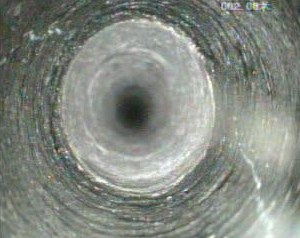

Supplement: S2 File — (ZIP) [file pone.0199749.s002.zip › S2_File/(1).tif]

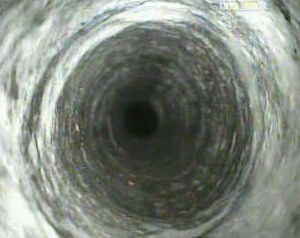

Supplement: S2 File — (ZIP) [file pone.0199749.s002.zip › S2_File/(10).tif]

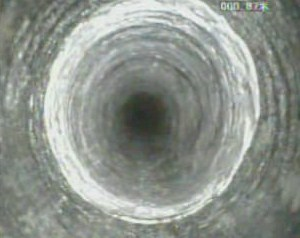

Supplement: S2 File — (ZIP) [file pone.0199749.s002.zip › S2_File/(11).tif]

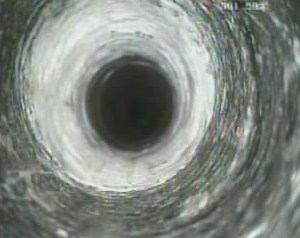

Supplement: S2 File — (ZIP) [file pone.0199749.s002.zip › S2_File/(12).tif]

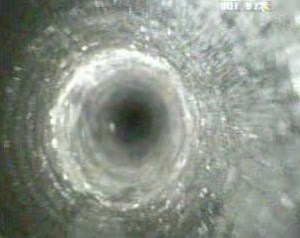

Supplement: S2 File — (ZIP) [file pone.0199749.s002.zip › S2_File/(13).tif]

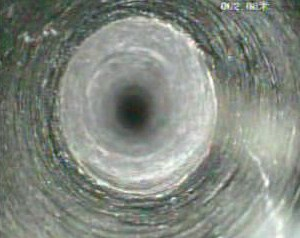

Supplement: S2 File — (ZIP) [file pone.0199749.s002.zip › S2_File/(14).tif]

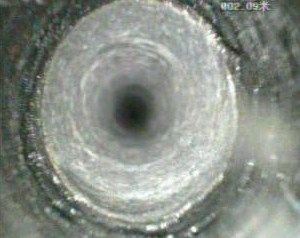

Supplement: S2 File — (ZIP) [file pone.0199749.s002.zip › S2_File/(15).tif]

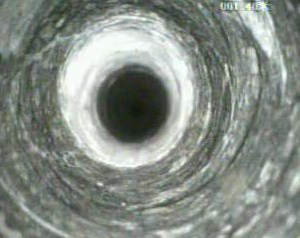

Supplement: S2 File — (ZIP) [file pone.0199749.s002.zip › S2_File/(16).tif]

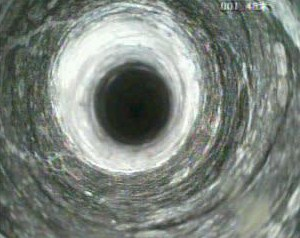

Supplement: S2 File — (ZIP) [file pone.0199749.s002.zip › S2_File/(17).tif]

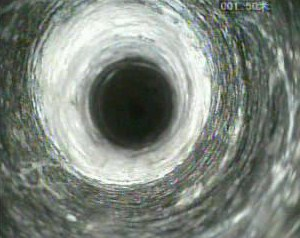

Supplement: S2 File — (ZIP) [file pone.0199749.s002.zip › S2_File/(18).tif]

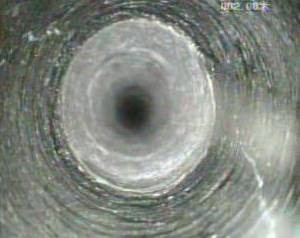

Supplement: S2 File — (ZIP) [file pone.0199749.s002.zip › S2_File/(19).tif]

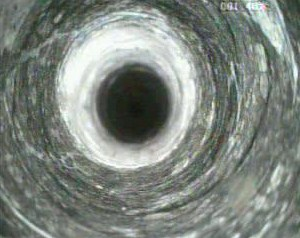

Supplement: S2 File — (ZIP) [file pone.0199749.s002.zip › S2_File/(2).tif]

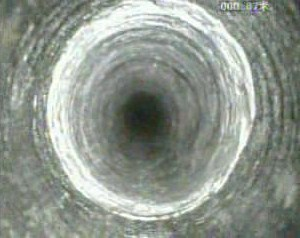

Supplement: S2 File — (ZIP) [file pone.0199749.s002.zip › S2_File/(20).tif]

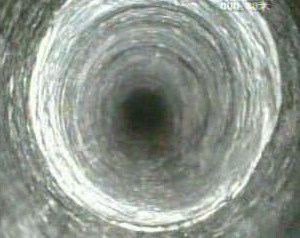

Supplement: S2 File — (ZIP) [file pone.0199749.s002.zip › S2_File/(21).tif]

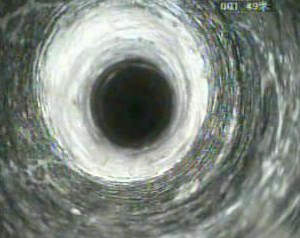

Supplement: S2 File — (ZIP) [file pone.0199749.s002.zip › S2_File/(22).tif]

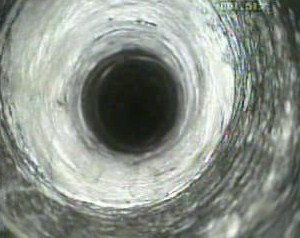

Supplement: S2 File — (ZIP) [file pone.0199749.s002.zip › S2_File/(23).tif]

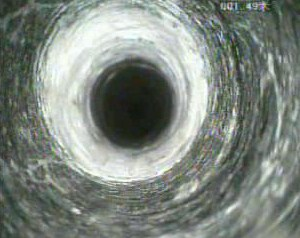

Supplement: S2 File — (ZIP) [file pone.0199749.s002.zip › S2_File/(24).tif]

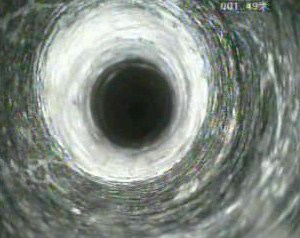

Supplement: S2 File — (ZIP) [file pone.0199749.s002.zip › S2_File/(25).tif]

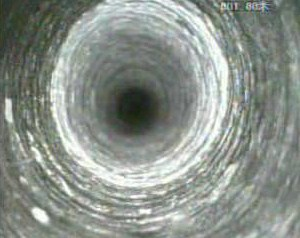

Supplement: S2 File — (ZIP) [file pone.0199749.s002.zip › S2_File/(26).tif]

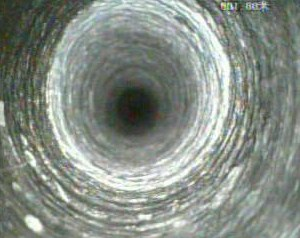

Supplement: S2 File — (ZIP) [file pone.0199749.s002.zip › S2_File/(27).tif]

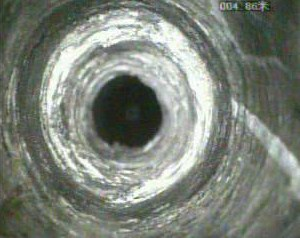

Supplement: S2 File — (ZIP) [file pone.0199749.s002.zip › S2_File/(28).tif]

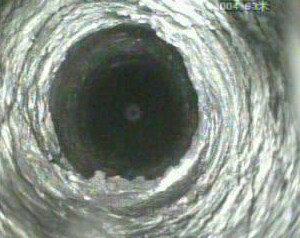

Supplement: S2 File — (ZIP) [file pone.0199749.s002.zip › S2_File/(29).tif]

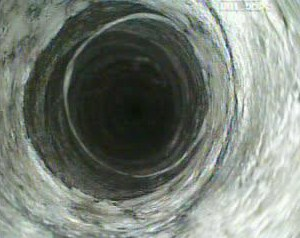

Supplement: S2 File — (ZIP) [file pone.0199749.s002.zip › S2_File/(3).tif]

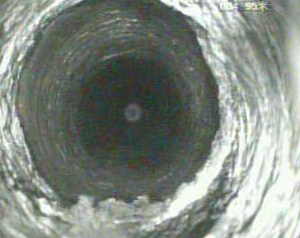

Supplement: S2 File — (ZIP) [file pone.0199749.s002.zip › S2_File/(30).tif]

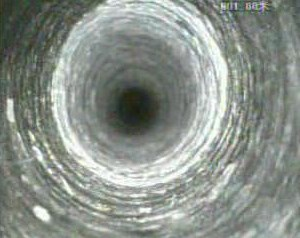

Supplement: S2 File — (ZIP) [file pone.0199749.s002.zip › S2_File/(31).tif]

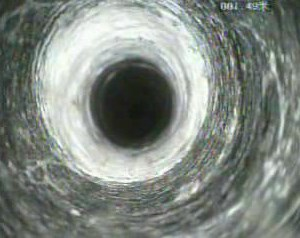

Supplement: S2 File — (ZIP) [file pone.0199749.s002.zip › S2_File/(32).tif]

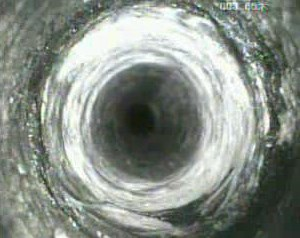

Supplement: S2 File — (ZIP) [file pone.0199749.s002.zip › S2_File/(33).tif]

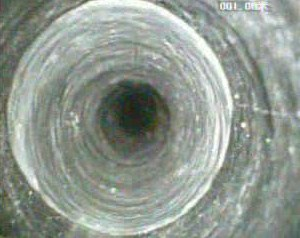

Supplement: S2 File — (ZIP) [file pone.0199749.s002.zip › S2_File/(34).tif]

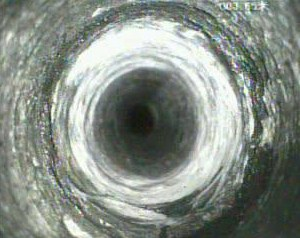

Supplement: S2 File — (ZIP) [file pone.0199749.s002.zip › S2_File/(35).tif]

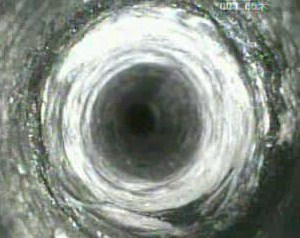

Supplement: S2 File — (ZIP) [file pone.0199749.s002.zip › S2_File/(36).tif]

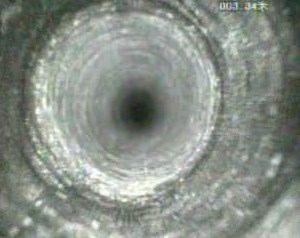

Supplement: S2 File — (ZIP) [file pone.0199749.s002.zip › S2_File/(37).tif]

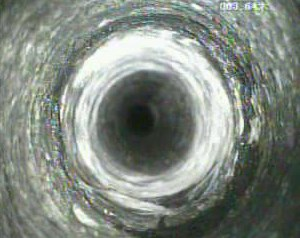

Supplement: S2 File — (ZIP) [file pone.0199749.s002.zip › S2_File/(38).tif]

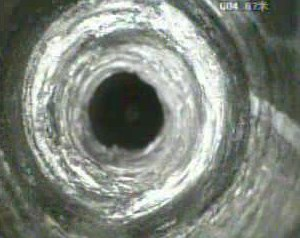

Supplement: S2 File — (ZIP) [file pone.0199749.s002.zip › S2_File/(39).tif]

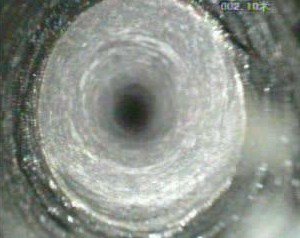

Supplement: S2 File — (ZIP) [file pone.0199749.s002.zip › S2_File/(4).tif]

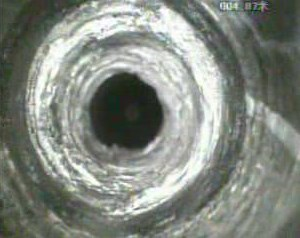

Supplement: S2 File — (ZIP) [file pone.0199749.s002.zip › S2_File/(40).tif]

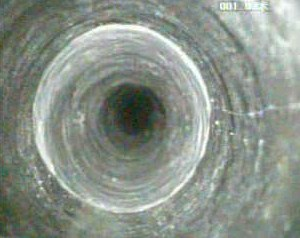

Supplement: S2 File — (ZIP) [file pone.0199749.s002.zip › S2_File/(41).tif]

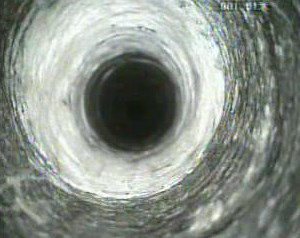

Supplement: S2 File — (ZIP) [file pone.0199749.s002.zip › S2_File/(42).tif]

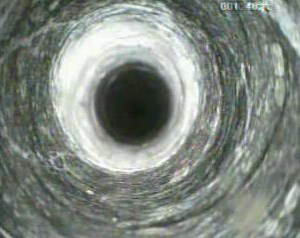

Supplement: S2 File — (ZIP) [file pone.0199749.s002.zip › S2_File/(43).tif]

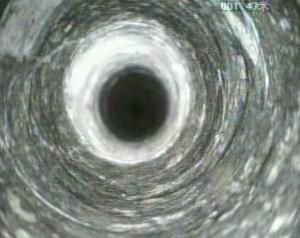

Supplement: S2 File — (ZIP) [file pone.0199749.s002.zip › S2_File/(44).tif]

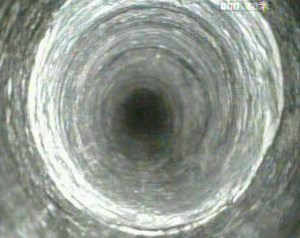

Supplement: S2 File — (ZIP) [file pone.0199749.s002.zip › S2_File/(45).tif]

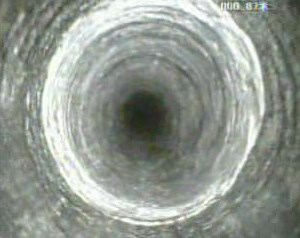

Supplement: S2 File — (ZIP) [file pone.0199749.s002.zip › S2_File/(46).tif]

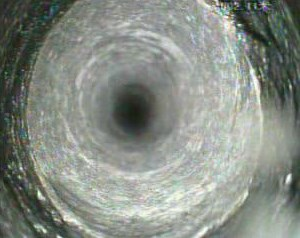

Supplement: S2 File — (ZIP) [file pone.0199749.s002.zip › S2_File/(47).tif]

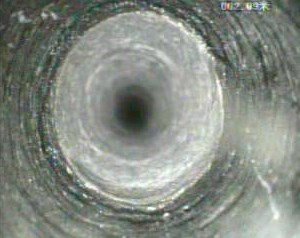

Supplement: S2 File — (ZIP) [file pone.0199749.s002.zip › S2_File/(48).tif]

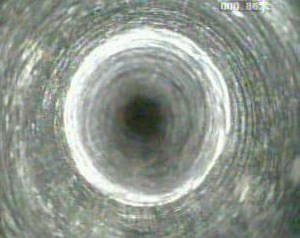

Supplement: S2 File — (ZIP) [file pone.0199749.s002.zip › S2_File/(49).tif]

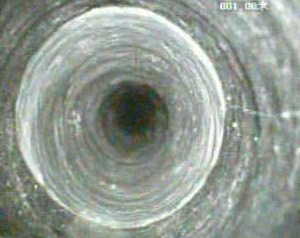

Supplement: S2 File — (ZIP) [file pone.0199749.s002.zip › S2_File/(50).tif]

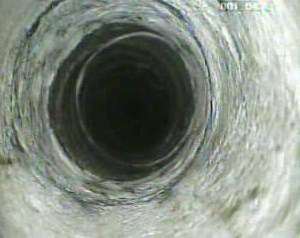

Supplement: S2 File — (ZIP) [file pone.0199749.s002.zip › S2_File/(6).tif]

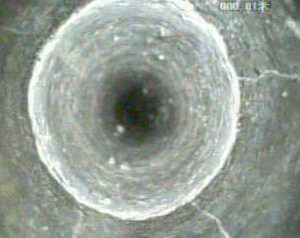

Supplement: S2 File — (ZIP) [file pone.0199749.s002.zip › S2_File/(7).tif]

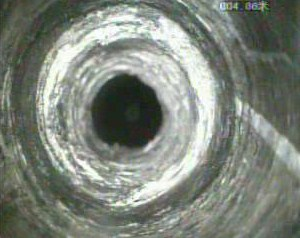

Supplement: S2 File — (ZIP) [file pone.0199749.s002.zip › S2_File/(8).tif]

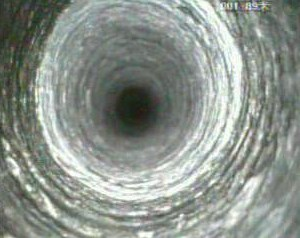

Supplement: S2 File — (ZIP) [file pone.0199749.s002.zip › S2_File/(9).tif]

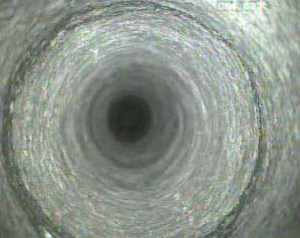

Supplement: S3 File — (ZIP) [file pone.0199749.s003.zip › S3_File/(1).tif]

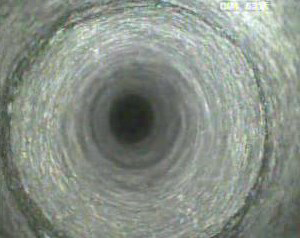

Supplement: S3 File — (ZIP) [file pone.0199749.s003.zip › S3_File/(10).tif]

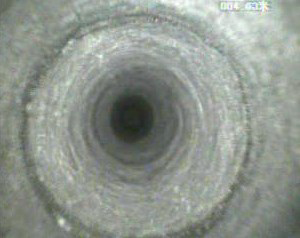

Supplement: S3 File — (ZIP) [file pone.0199749.s003.zip › S3_File/(11).tif]

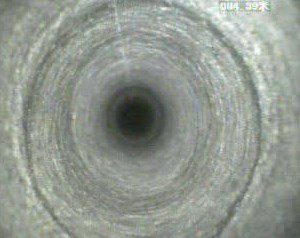

Supplement: S3 File — (ZIP) [file pone.0199749.s003.zip › S3_File/(12).tif]

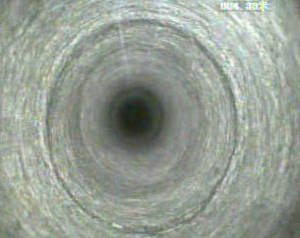

Supplement: S3 File — (ZIP) [file pone.0199749.s003.zip › S3_File/(13).tif]

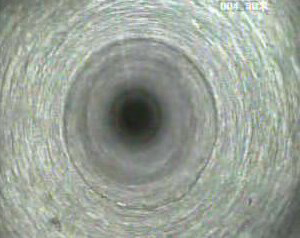

Supplement: S3 File — (ZIP) [file pone.0199749.s003.zip › S3_File/(14).tif]

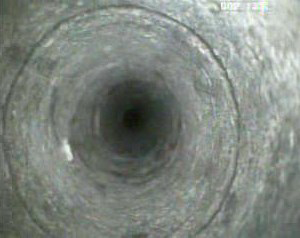

Supplement: S3 File — (ZIP) [file pone.0199749.s003.zip › S3_File/(15).tif]

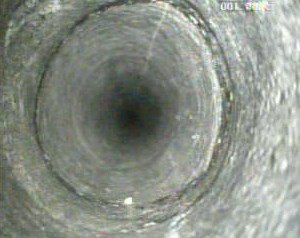

Supplement: S3 File — (ZIP) [file pone.0199749.s003.zip › S3_File/(16).tif]

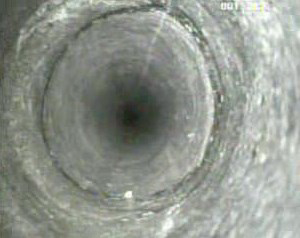

Supplement: S3 File — (ZIP) [file pone.0199749.s003.zip › S3_File/(17).tif]

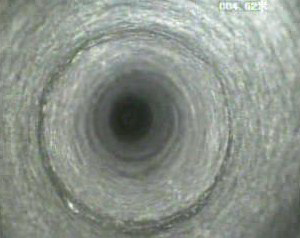

Supplement: S3 File — (ZIP) [file pone.0199749.s003.zip › S3_File/(18).tif]

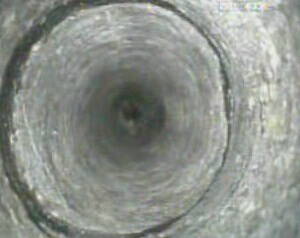

Supplement: S3 File — (ZIP) [file pone.0199749.s003.zip › S3_File/(19).tif]

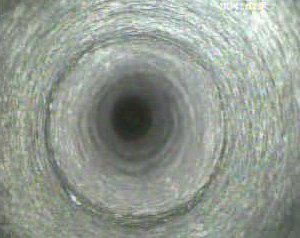

Supplement: S3 File — (ZIP) [file pone.0199749.s003.zip › S3_File/(2).tif]

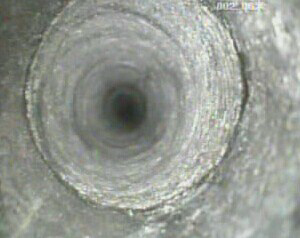

Supplement: S3 File — (ZIP) [file pone.0199749.s003.zip › S3_File/(20).tif]

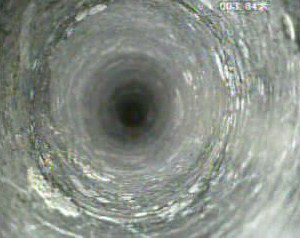

Supplement: S3 File — (ZIP) [file pone.0199749.s003.zip › S3_File/(21).tif]

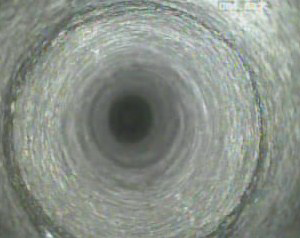

Supplement: S3 File — (ZIP) [file pone.0199749.s003.zip › S3_File/(22).tif]

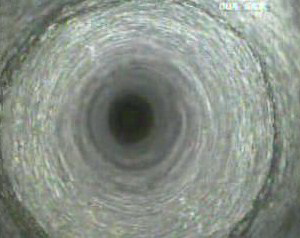

Supplement: S3 File — (ZIP) [file pone.0199749.s003.zip › S3_File/(23).tif]

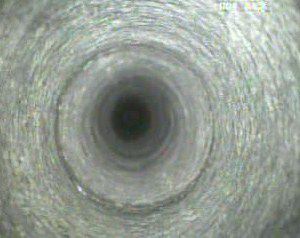

Supplement: S3 File — (ZIP) [file pone.0199749.s003.zip › S3_File/(24).tif]

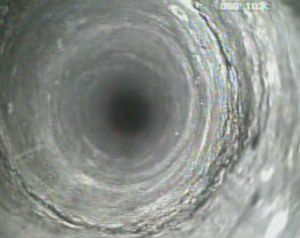

Supplement: S3 File — (ZIP) [file pone.0199749.s003.zip › S3_File/(25).tif]

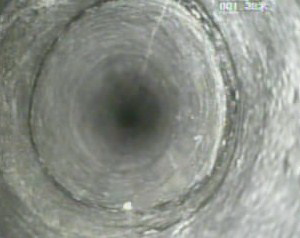

Supplement: S3 File — (ZIP) [file pone.0199749.s003.zip › S3_File/(26).tif]

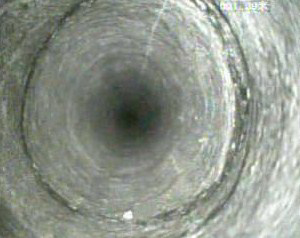

Supplement: S3 File — (ZIP) [file pone.0199749.s003.zip › S3_File/(27).tif]

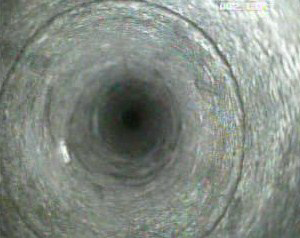

Supplement: S3 File — (ZIP) [file pone.0199749.s003.zip › S3_File/(28).tif]

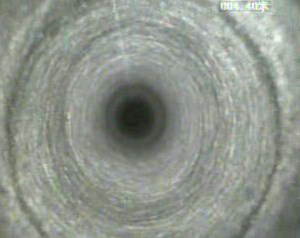

Supplement: S3 File — (ZIP) [file pone.0199749.s003.zip › S3_File/(3).tif]

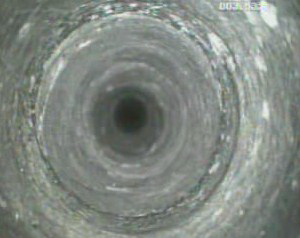

Supplement: S3 File — (ZIP) [file pone.0199749.s003.zip › S3_File/(31).tif]

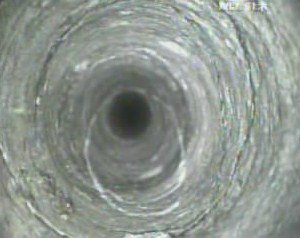

Supplement: S3 File — (ZIP) [file pone.0199749.s003.zip › S3_File/(32).tif]

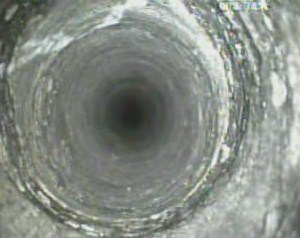

Supplement: S3 File — (ZIP) [file pone.0199749.s003.zip › S3_File/(33).tif]

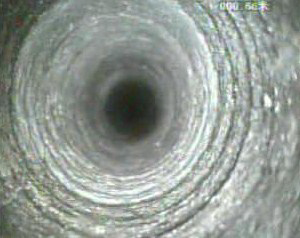

Supplement: S3 File — (ZIP) [file pone.0199749.s003.zip › S3_File/(35).tif]

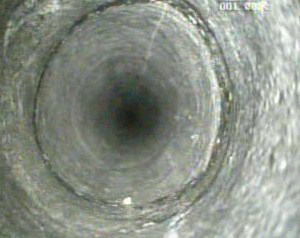

Supplement: S3 File — (ZIP) [file pone.0199749.s003.zip › S3_File/(36).tif]

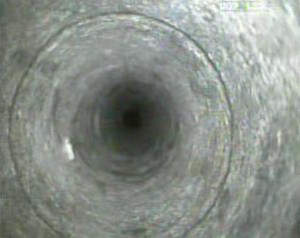

Supplement: S3 File — (ZIP) [file pone.0199749.s003.zip › S3_File/(38).tif]

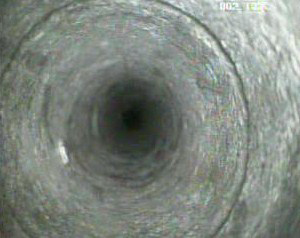

Supplement: S3 File — (ZIP) [file pone.0199749.s003.zip › S3_File/(40).tif]

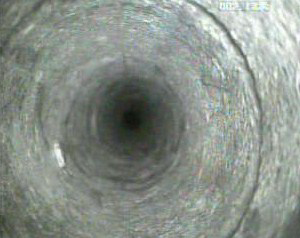

Supplement: S3 File — (ZIP) [file pone.0199749.s003.zip › S3_File/(41).tif]

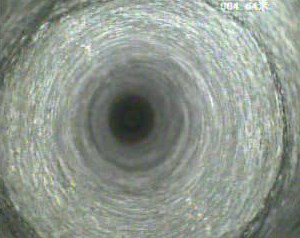

Supplement: S3 File — (ZIP) [file pone.0199749.s003.zip › S3_File/(48).tif]

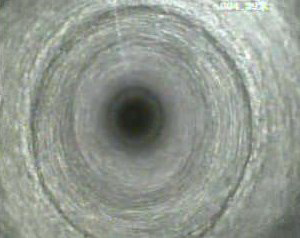

Supplement: S3 File — (ZIP) [file pone.0199749.s003.zip › S3_File/(5).tif]

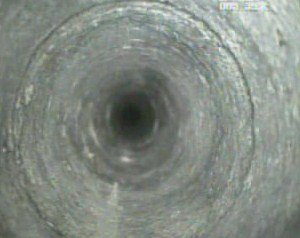

Supplement: S3 File — (ZIP) [file pone.0199749.s003.zip › S3_File/(50).tif]

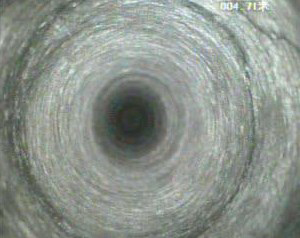

Supplement: S3 File — (ZIP) [file pone.0199749.s003.zip › S3_File/(6).tif]

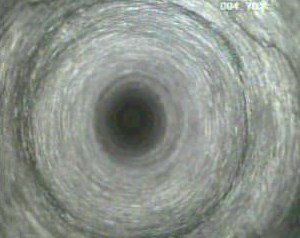

Supplement: S3 File — (ZIP) [file pone.0199749.s003.zip › S3_File/(7).tif]

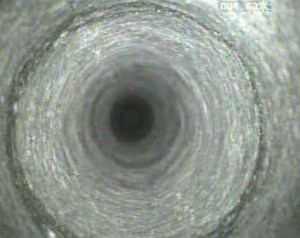

Supplement: S3 File — (ZIP) [file pone.0199749.s003.zip › S3_File/(8).tif]

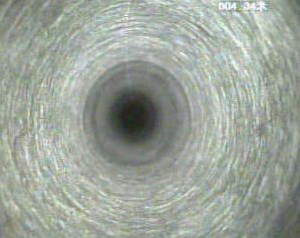

Supplement: S4 File — (ZIP) [file pone.0199749.s004.zip › S4_File/(1).tif]

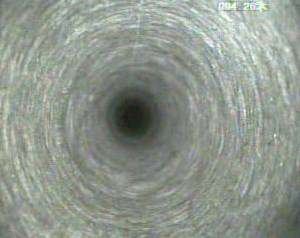

Supplement: S4 File — (ZIP) [file pone.0199749.s004.zip › S4_File/(10).tif]

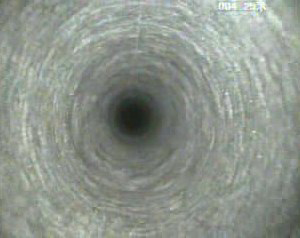

Supplement: S4 File — (ZIP) [file pone.0199749.s004.zip › S4_File/(11).tif]

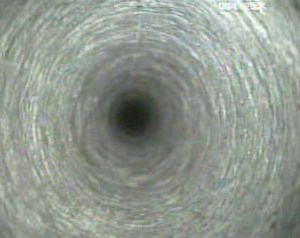

Supplement: S4 File — (ZIP) [file pone.0199749.s004.zip › S4_File/(12).tif]

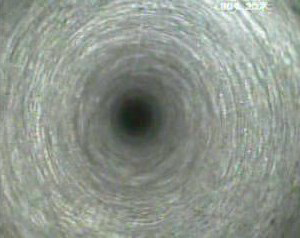

Supplement: S4 File — (ZIP) [file pone.0199749.s004.zip › S4_File/(13).tif]

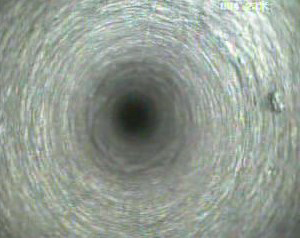

Supplement: S4 File — (ZIP) [file pone.0199749.s004.zip › S4_File/(14).tif]

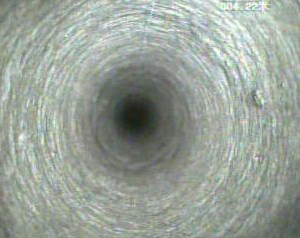

Supplement: S4 File — (ZIP) [file pone.0199749.s004.zip › S4_File/(15).tif]

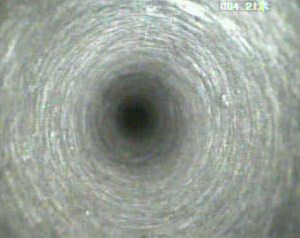

Supplement: S4 File — (ZIP) [file pone.0199749.s004.zip › S4_File/(16).tif]

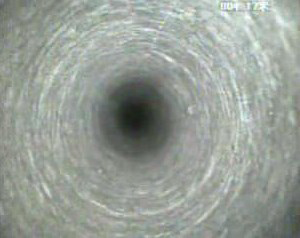

Supplement: S4 File — (ZIP) [file pone.0199749.s004.zip › S4_File/(17).tif]

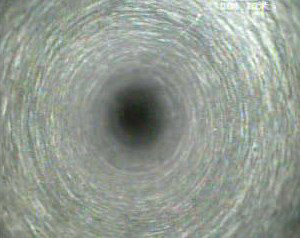

Supplement: S4 File — (ZIP) [file pone.0199749.s004.zip › S4_File/(18).tif]

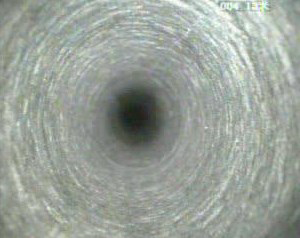

Supplement: S4 File — (ZIP) [file pone.0199749.s004.zip › S4_File/(19).tif]

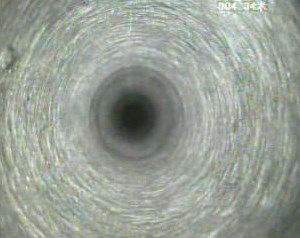

Supplement: S4 File — (ZIP) [file pone.0199749.s004.zip › S4_File/(2).tif]

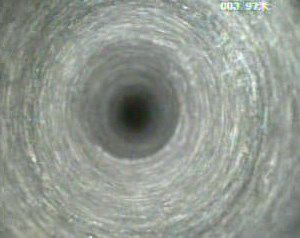

Supplement: S4 File — (ZIP) [file pone.0199749.s004.zip › S4_File/(20).tif]

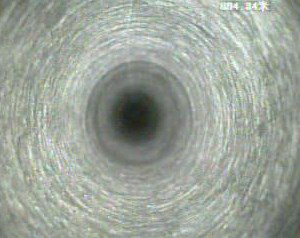

Supplement: S4 File — (ZIP) [file pone.0199749.s004.zip › S4_File/(21).tif]

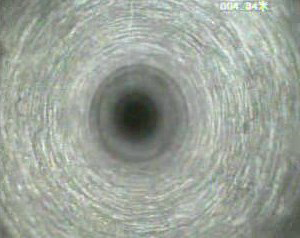

Supplement: S4 File — (ZIP) [file pone.0199749.s004.zip › S4_File/(22).tif]
