# Supplementary figures and images for: A two-stage classification method for borehole-wall images with support vector machine (part 2 of 2)
Source: PLoS One. 2018 Jun 28;13(6):e0199749. doi: 10.1371/journal.pone.0199749 (PMC6023159; doi:10.1371/journal.pone.0199749)

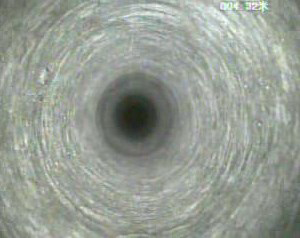

Supplement: S4 File — (ZIP) [file pone.0199749.s004.zip › S4_File/(23).tif]

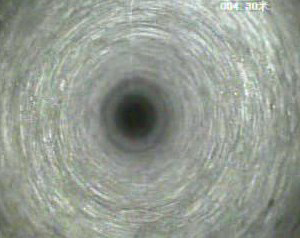

Supplement: S4 File — (ZIP) [file pone.0199749.s004.zip › S4_File/(24).tif]

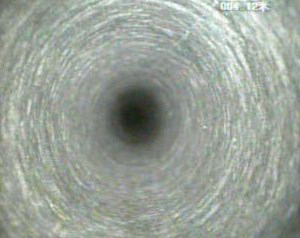

Supplement: S4 File — (ZIP) [file pone.0199749.s004.zip › S4_File/(25).tif]

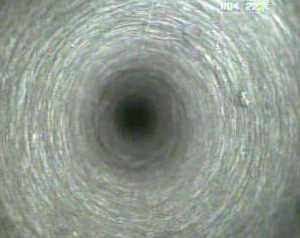

Supplement: S4 File — (ZIP) [file pone.0199749.s004.zip › S4_File/(26).tif]

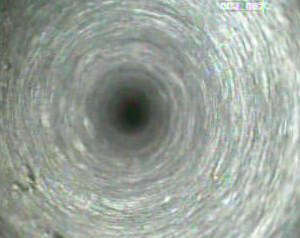

Supplement: S4 File — (ZIP) [file pone.0199749.s004.zip › S4_File/(27).tif]

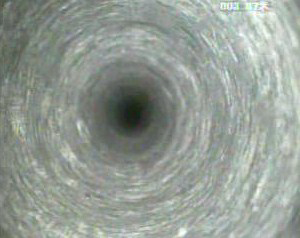

Supplement: S4 File — (ZIP) [file pone.0199749.s004.zip › S4_File/(28).tif]

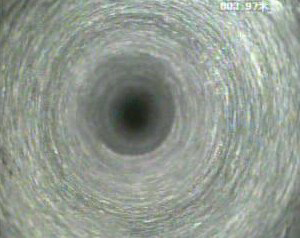

Supplement: S4 File — (ZIP) [file pone.0199749.s004.zip › S4_File/(29).tif]

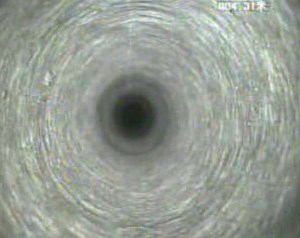

Supplement: S4 File — (ZIP) [file pone.0199749.s004.zip › S4_File/(3).tif]

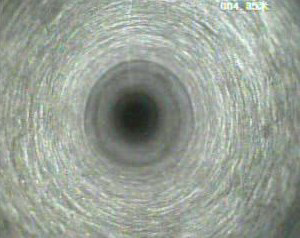

Supplement: S4 File — (ZIP) [file pone.0199749.s004.zip › S4_File/(30).tif]

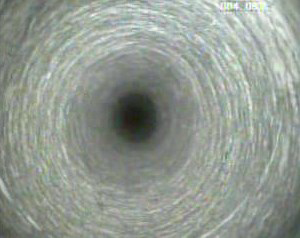

Supplement: S4 File — (ZIP) [file pone.0199749.s004.zip › S4_File/(31).tif]

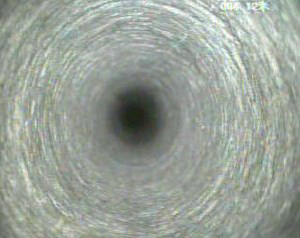

Supplement: S4 File — (ZIP) [file pone.0199749.s004.zip › S4_File/(32).tif]

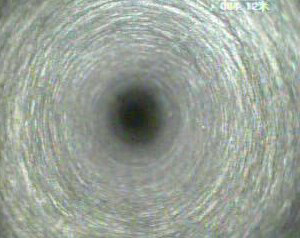

Supplement: S4 File — (ZIP) [file pone.0199749.s004.zip › S4_File/(33).tif]

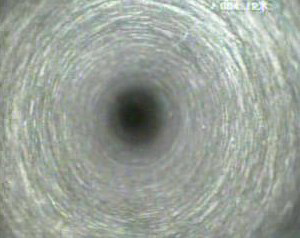

Supplement: S4 File — (ZIP) [file pone.0199749.s004.zip › S4_File/(34).tif]

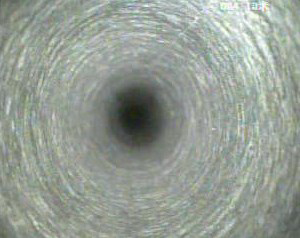

Supplement: S4 File — (ZIP) [file pone.0199749.s004.zip › S4_File/(36).tif]

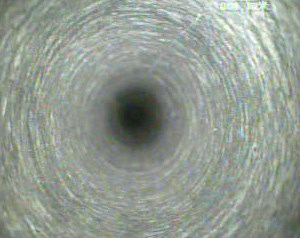

Supplement: S4 File — (ZIP) [file pone.0199749.s004.zip › S4_File/(37).tif]

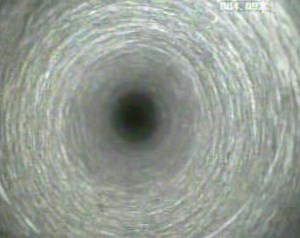

Supplement: S4 File — (ZIP) [file pone.0199749.s004.zip › S4_File/(38).tif]

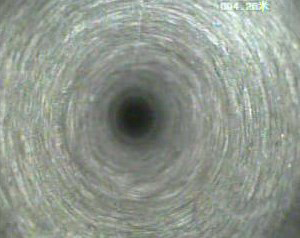

Supplement: S4 File — (ZIP) [file pone.0199749.s004.zip › S4_File/(39).tif]

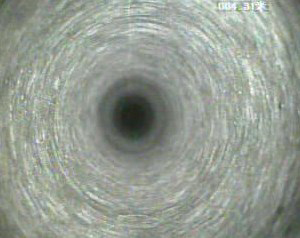

Supplement: S4 File — (ZIP) [file pone.0199749.s004.zip › S4_File/(4).tif]

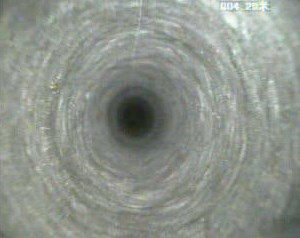

Supplement: S4 File — (ZIP) [file pone.0199749.s004.zip › S4_File/(40).tif]

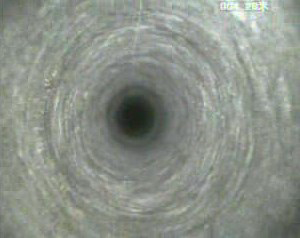

Supplement: S4 File — (ZIP) [file pone.0199749.s004.zip › S4_File/(41).tif]

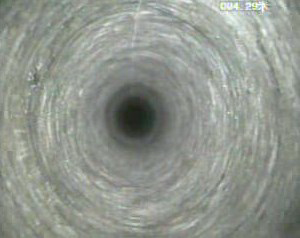

Supplement: S4 File — (ZIP) [file pone.0199749.s004.zip › S4_File/(42).tif]

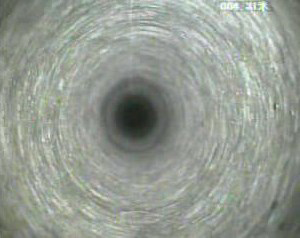

Supplement: S4 File — (ZIP) [file pone.0199749.s004.zip › S4_File/(43).tif]

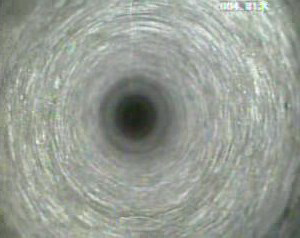

Supplement: S4 File — (ZIP) [file pone.0199749.s004.zip › S4_File/(44).tif]

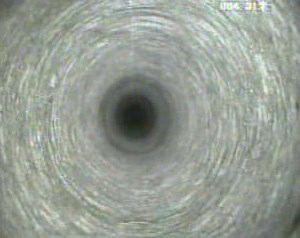

Supplement: S4 File — (ZIP) [file pone.0199749.s004.zip › S4_File/(45).tif]

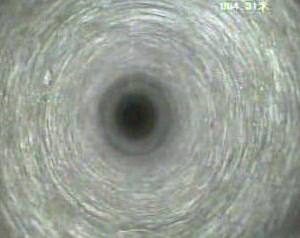

Supplement: S4 File — (ZIP) [file pone.0199749.s004.zip › S4_File/(47).tif]

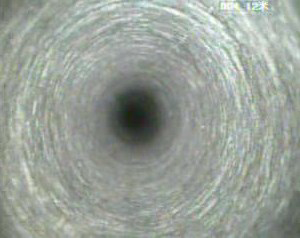

Supplement: S4 File — (ZIP) [file pone.0199749.s004.zip › S4_File/(48).tif]

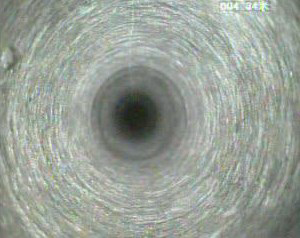

Supplement: S4 File — (ZIP) [file pone.0199749.s004.zip › S4_File/(49).tif]

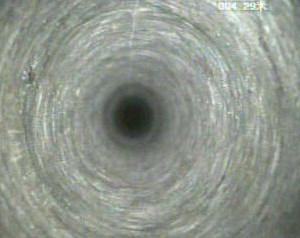

Supplement: S4 File — (ZIP) [file pone.0199749.s004.zip › S4_File/(5).tif]

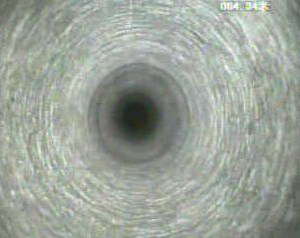

Supplement: S4 File — (ZIP) [file pone.0199749.s004.zip › S4_File/(50).tif]

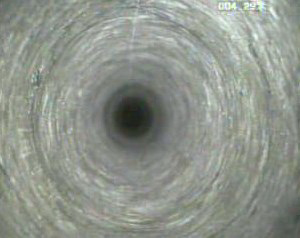

Supplement: S4 File — (ZIP) [file pone.0199749.s004.zip › S4_File/(6).tif]

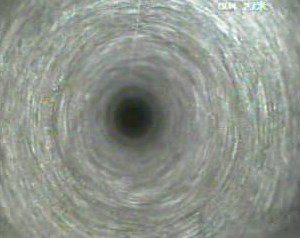

Supplement: S4 File — (ZIP) [file pone.0199749.s004.zip › S4_File/(7).tif]

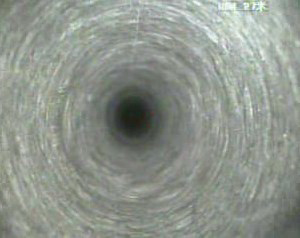

Supplement: S4 File — (ZIP) [file pone.0199749.s004.zip › S4_File/(8).tif]

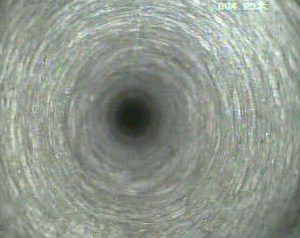

Supplement: S4 File — (ZIP) [file pone.0199749.s004.zip › S4_File/(9).tif]
